# The anti-tumor efficacy of 3C23K, a glyco-engineered humanized anti-MISRII antibody, in an ovarian cancer model is mainly mediated by engagement of immune effector cells

**DOI:** 10.18632/oncotarget.15715

**Published:** 2017-02-24

**Authors:** Pauline Estupina, Alexandre Fontayne, Jean-Marc Barret, Nathalie Kersual, Olivier Dubreuil, Marion Le Blay, Alexandre Pichard, Marta Jarlier, Martine Pugnière, Maëva Chauvin, Thierry Chardès, Jean-Pierre Pouget, Emmanuel Deshayes, Alexis Rossignol, Toufik Abache, Christophe de Romeuf, Aurélie Terrier, Lucie Verhaeghe, Christine Gaucher, Jean-François Prost, André Pèlegrin, Isabelle Navarro-Teulon

**Affiliations:** ^1^ IRCM, Institut de Recherche en Cancérologie de Montpellier, Montpellier, F-34298, France; ^2^ INSERM, U896, Montpellier, F-34298, France; ^3^ Université Montpellier, Montpellier, F-34298, France; ^4^ Institut Régional du Cancer de Montpellier, ICM, Montpellier, F-34298, France; ^5^ LFB Biotechnologies, Loos, F-59120, France; ^6^ GamaMabs Pharma, Toulouse cedex, F-31106, France; ^7^ Clean Cells, Boufféré, F-85600, France

**Keywords:** immunotherapy, therapeutic antibody, ovarian cancer, GCT, MISRII

## Abstract

Ovarian cancer is the leading cause of death in women with gynecological cancers and despite recent advances, new and more efficient therapies are crucially needed. Müllerian Inhibiting Substance type II Receptor (MISRII, also named AMHRII) is expressed in most ovarian cancer subtypes and is a novel potential target for ovarian cancer immunotherapy. We previously developed and tested 12G4, the first murine monoclonal antibody (MAb) against human MISRII. Here, we report the humanization, affinity maturation and glyco-engineering steps of 12G4 to generate the Fc-optimized 3C23K MAb, and the evaluation of its *in vivo* anti-tumor activity. The epitopes of 3C23K and 12G4 were strictly identical and 3C23K affinity for MISRII was enhanced by a factor of about 14 (K_D_ = 5.5 × 10^−11^ M vs 7.9 × 10^−10^ M), while the use of the EMABling^®^ platform allowed the production of a low-fucosylated 3C23K antibody with a 30-fold K_D_ improvement of its affinity to FcγRIIIa. In COV434-MISRII tumor-bearing mice, 3C23K reduced tumor growth more efficiently than 12G4 and its combination with carboplatin was more efficient than each monotherapy with a mean tumor size of 500, 1100 and 100 mm^3^ at the end of treatment with 3C23K (10 mg/kg, Q3-4D12), carboplatin (60 mg/kg, Q7D4) and 3C23K+carboplatin, respectively. Conversely, 3C23K-FcKO, a 3C23K form without affinity for the FcγRIIIa receptor, did not display any anti-tumor effect *in vivo*. These results strongly suggested that 3C23K mechanisms of action are mainly Fc-related. *In vitro*, antibody-dependent cytotoxicity (ADCC) and antibody-dependent cell phagocytosis (ADCP) were induced by 3C23K, as demonstrated with human effector cells. Using human NK cells, 50% of the maximal lysis was obtained with a 46-fold lower concentration of low-fucosylated 3C23K (2.9 ng/ml) than of 3C23K expressed in CHO cells (133.35 ng/ml). As 3C23K induced strong ADCC with human PBMC but almost none with murine PBMC, antibody-dependent cell phagocytosis (ADCP) was then investigated. 3C23K-dependent (100 ng/ml) ADCP was more active with murine than human macrophages (only 10% of living target cells *vs*. about 25%). These *in vitro* results suggest that the reduced ADCC with murine effectors could be partially balanced by ADCP activity in *in vivo* experiments. Taken together, these preclinical data indicate that 3C23K is a new promising therapeutic candidate for ovarian cancer immunotherapy and justify its recent introduction in a phase I clinical trial.

## INTRODUCTION

Ovarian cancer (OC) accounts for 3–5% of all cancers in women and is the leading cause of death in women with gynecological cancer [1, 2]. The relative absence of specific signs and symptoms, coupled with the lack of reliable screening strategies, makes of OC a “silent killer”. Indeed, it is often diagnosed at advanced stages when the disease has already spread to the abdominal cavity (over 70% at stage III or IV), thus resulting in low cure rates. The outcome for women with OC is generally poor, with an overall five year survival rate of 40% [3].

The current standard of care for OC involves a combination of debulking surgery and platinum-based chemotherapy (carboplatin combined with paclitaxel is the most effective chemotherapeutic regimen). Many women respond well initially to this therapeutic approach; however, 70 to 90% eventually will develop chemo-resistant recurrent OC [3].

Thus, to improve the clinical prognosis, new OC therapies are crucially needed. Various targeted strategies have been evaluated for OC management [1, 4, 5] and many molecules are under investigation, among which agents to block growth factor receptors, such as monoclonal antibodies (MAbs), or tyrosine kinase inhibitors (TKIs), anti-angiogenetic molecules and DNA repair inhibitors, such as Poly(ADP-ribose) polymerase (PARP) inhibitors such olaparib (for review see [5]). However, these molecules only provide short-term survival improvement [6] and more effective treatments to address chemo-resistant recurrent OC are not available yet. MAbs against surface antigens that are effective in different cancer types [7, 8] display no or extremely moderate activities when tested in OCs. Nevertheless, a few innovative targets, among which folate receptor [9] or angiopoietins [10], are currently evaluated. Finally, OC heterogeneity also complicates the targeted treatment strategies. The molecules evaluated in clinical trials do not seem to be clinically effective and with low toxicity in OCs [4]. Recently, in phase 2 trials, eight targeted drugs (gefitinib, imatinib, sorafenib, temsirolimus, mifepristone, enzastaurine, lapatinib and vorinostat) have produced objective response rates of less than 10% and stabilized the disease for six months in less than 25% of patients [11]. Moreover, besides olaparib and bevacizumab, no new therapy for OC has been approved in almost ten years [5]. New treatments are, therefore, required to improve OC outcome.

Müllerian Inhibiting Substance type II Receptor (MISRII, also named AMHRII) is a novel potential target for OC immunotherapy. The Müllerian Inhibiting Substance (MIS)/MISRII pathway plays a crucial role in Müllerian duct regression in males during fetal development. MISRII is a member of the TGF-β family (< 30% homology with the other members) and has no homology with other human proteins [12]. MISRII expression profile in normal tissues is related to its involvement in gonad development and functions. Indeed, it is mainly expressed in granulosa cells of the ovary and in Sertoli and Leydig cells of the testis [13]. However, it is also detected in rodent uterus as well as in human endometrium, breast and prostate tissues and, surprisingly, in brain motor neurons [13–15]. MISRII is expressed in most human granulosa cell tumors (GCT), a rare OC form [16], and in the majority of human epithelial OCs (EOC) [17–20] as well as in the derived ascites, but only for a limited period of time [21]. Moreover, recombinant human MIS can inhibit the growth of mouse or human ovarian cancer cell lines *in vitro* and *in vivo* [22–24]. Therefore, MISRII represent a new candidate for targeted therapy in OC.

We developed and characterized 12G4, the first murine MAb against human MISRII [25]. This antibody showed good anti-tumor efficacy *in vitro* and *in vivo* using two OC xenograft models (NIH-OvCar3 and COV434-MISRII cells derived from human OCs) [20]. These findings confirm that anti-MISRII immunotherapy represents a new promising approach for treating MISRII-positive OCs (especially GCT and EOC) and that the MAb 12G4 could be an attractive candidate to efficiently target this receptor. However, in the clinical practice, a mouse MAb could elicit human anti-murine antibody (HAMA) responses in patients [26]. Therefore, the aim of this study was to generate a humanized version of 12G4 and to demonstrate its activity against ovarian cancer cells *in vitro* and *in vivo*. Chimerization, complementarity determining region (CDR) grafting and molecular evolution were used to generate the humanized 3C23K MAb starting from 12G4. Moreover, to restore the binding affinity that was partially lost during the initial CDR grafting step, the framework (FR) sequences of the variable region were fine-tuned by using phage-displayed combinatorial libraries [27]. The EMABling^®^ technology was also used to produce a low-fucosylated 3C23K with the aim of increasing its antibody-dependent cell-mediated cytotoxicity (ADCC) and consequently its anti-tumor activity [28–30].

## RESULTS

### Humanization: from the murine MAb 12G4 to the humanized MAb 3C23K

### Chimerization, humanization and affinity maturation

The 3C23K humanized antibody was initially derived from the variable regions of the murine 12G4 MAb [25]. The humanization procedure included CDR grafting (MAb h12G4) and affinity maturation by random mutagenesis and phage display, leading to the final molecule 3C23K.

In the first step, candidate human templates for CDR grafting were identified by separately entering the sequences of the VL and VH domains in the IMGT/DomainGapAlign search program [31] and by restricting the search to human sequences in IMGT/GENE-DB [32]. The closest human VH gene, IGHV1-3*01, showed 67.34% of identity with the murine counterpart. This identity rose up to 92.85% after grafting the murine 12G4 CDR-IMGT into the human FR-IMGT. The closest human VL gene, IGK1-9*01, showed 62.76% of identity with the murine counterpart. However, IGKV1-5*01 was preferred because the IMGT/GeneFrequency tool [31] indicated that IGK1-9*01 is not very frequently expressed. IGKV1-5*01 has an identity of 58.51% with the VL of 12G4 that was increased to 88.29% after grafting.

**Figure 1:**
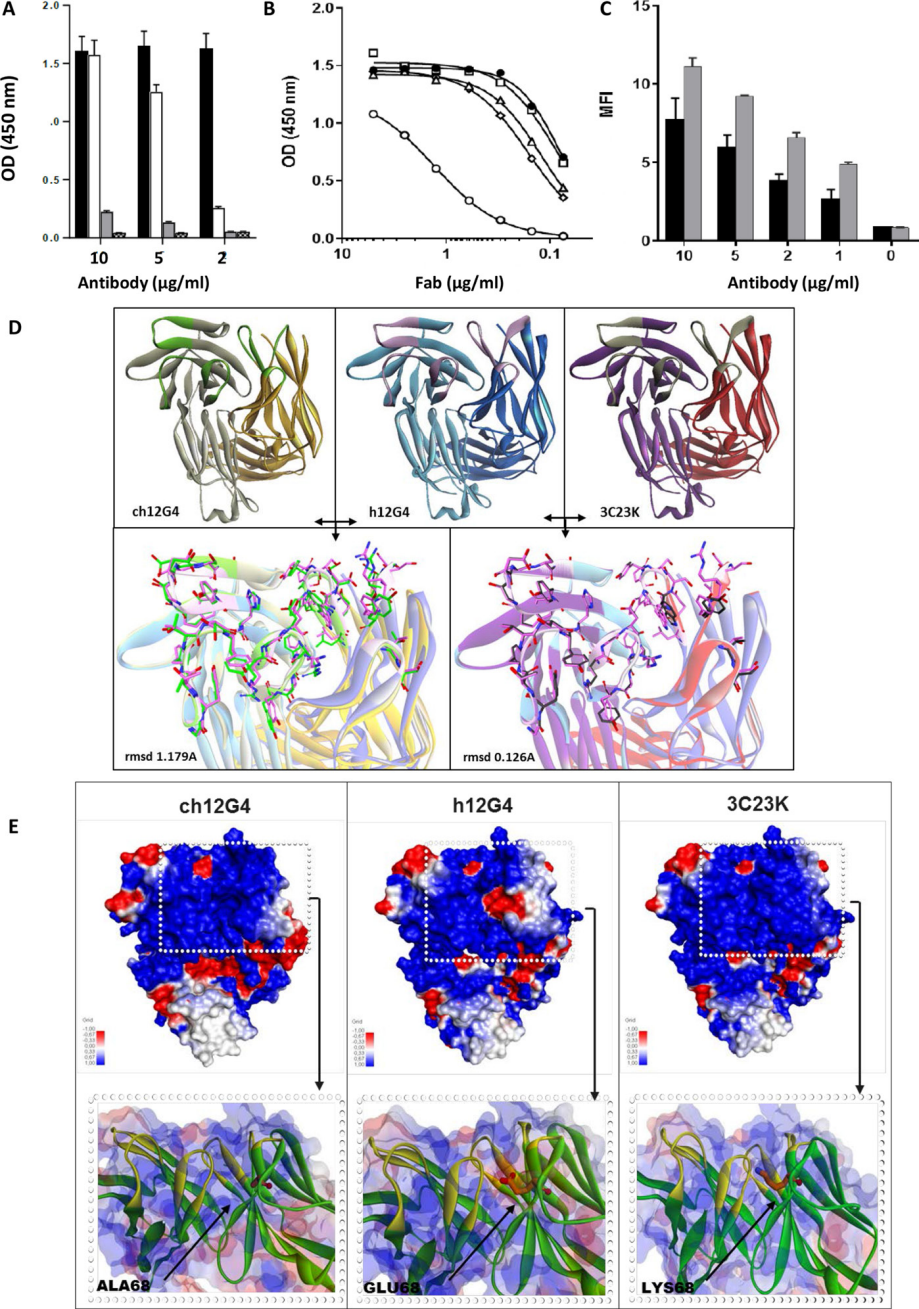
Humanization of the murine MAb 12G4 Panel (**A**) Comparison of the binding capacity of murine and humanized 12G4. In ELISA, MISRII-Fc was coated on titration plates and then antibodies were added at different concentrations before detection with the appropriate HRP-labeled secondary antibody. Black bars: murine 12G4; white bars: humanized 12G4 (h12G4), gray bars: uncoated control with murine 12G4; dashed bars uncoated control with h12G4. Panels B-C: Comparison of the binding capacity of humanized 12G4 (h12G4) and of the different affinity matured variants with that of murine 12G4. (**B**) In ELISA assays, microtiter plates were coated with MISRII-Fc and the tested Fabs were added at different concentrations before detection with an HRP-labeled secondary antibody. Black circles (●), murine 12G4; open circle(○), h12G4; open diamonds (◇), 6B78; open triangles (∆), 3C23; open squares (☐), 3C23K; (**C**) By cytometry analysis of COV434-MISRII cells using the antibodies 12G4 and 3C23K at 0, 1, 2, 5 or 10 μg/ml. Black bars: murine 12G4; Grey bars: 3C23K. Panels (D, E) Modeled structure of chimeric 12G4 (ch12G4), humanized 12G4 (h12G4) and affinity matured 3C23K using a sequence homology approach. Templates selected to build the initial model were the PDB structures 2OSL (for the light and heavy chains of ch12G4) and 3EO9 and 2EH7 (for the light and heavy chain of h12G4, respectively). (**D**) CDR loops, shown as sticks and with a different color than the light and heavy chains, were specifically rebuilt and refined using the Discovery Studio software (Modeler and Looper algorithms). The 3C23K model was built from the h12G4 model by replacing the four residues mutated during the maturation affinity process (I47T, S49P, E54K, Q216R showed as sticks and balls). The conformation of the mutated residues and of the surrounding residues that lie within a 5Å cutoff radius was optimized using the MODELER algorithm. (**E**) The electrostatic potentials of the three MAbs, calculated with the Delphi program included in the Discovery studio software, are visualized using a surface graphic view. Highly negative and highly positive regions are indicated in red and blue, respectively.

After production in YB2/0 cells and purification, the binding capacity of humanized 12G4 (h12G4) to the MISRII-Fc fusion protein was tested and compared with that of mouse 12G4 by ELISA (Figure 1A). The choice of a recombinant MISRII protein was based on the observation that MISRII expression in cell lines derived from OC and OC ascites rapidly and progressively decreases after long-term culture [21], thus limiting the result reproducibility. Compared with chimeric (ch12G4), the binding capacity of h12G4 was about 20-fold reduced. Therefore, to restore h12G4 binding affinity, both variable domains of the humanized antibody were modified using the MutaGen^™^ technology [27, 33], a random mutagenesis method. Based on the scFv format of h12G4, libraries were created and mixed before variant selection through six rounds of phage-display panning on immobilized recombinant MISRII-Fc fusion protein. Among the 113 clones selected by biopanning, 43 exhibited higher binding affinity than h12G4 by phage-ELISA assay (data not shown). These clones carried and/or shared mutations at positions known to affect the structural conformation of the CDR loops (I54T in the VL of clone 3C23), or the orientation of the V domains (L50P shared by the VH of clones 4C35 and 5B81). Other mutations were located inside the CDR-L2 loop (E68K shared by the VL of clones 4C35 and 6B78), or were modified by the humanization process (E68K in the VL of clones 4C35 and 6B78). To examine the real effect of these mutations independently of the scFv format, the corresponding VH and VL were subcloned in a bicistronic vector that allows the expression of Fab fragments in the periplasmic space of *E.coli* HB2151 cells. The Fab variants 6B78, 5B42, 4C35 and 3C23 showed a significant increase in binding affinity, compared with h12G4, in an ELISA assay with immobilized recombinant MISRII-Fc (Figure 1B and Supplementary Figure 1A and 1B). The clones 6B78 and 4C35 harbored the same mutation (E68K) in the VL, whereas clone 5B42 had a mutation in the VL (S56P). The mutation L50P (clone 5B81) had no effect on binding to recombinant MISRII-Fc (Supplementary Figure 1C). Despite the binding affinity improvement, none of the selected variants reached the binding capacity of the parental mouse antibody. Therefore, to further increase affinity, the mutation E68K (clones 6B78 and 4C35) was introduced in clone 3C23 to generate clone 3C23K that showed a binding affinity close to that of the parental 12G4 mouse antibody (Figure 1B). To better define the binding characteristics, clone 3C23K was reformatted as an IgG1 antibody, produced in YB2/0 cells and analyzed by surface plasmon resonance (SPR). The 3C23K antibody exhibited a higher binding affinity (K_D_ = 5.5 × 10^−11^ M) than mouse 12G4 (K_D_ = 7.9 × 10^−10^ M). This latter value was very close to the value published in the initial description of the MAb 12G4 (K_D_ = 8.6 × 10^−10^ M) [25]. The gain of binding affinity was also confirmed by flow cytometry using COV434-MISRII cells (Figure 1C).

### Epitope mapping of 3C23K

To analyze the epitopes recognized by 3C23K, 13 overlapping peptides of 20 amino acids each were produced. These peptides covered the first 132 amino acids of MISRII extracellular domain (Table 1). As positive control, a peptide centered on the epitope D^53^RAQVEM of the murine 12G4 MAb was used [25]. Peptides were immobilized on glass slides and incubated with 12G4 or 3C23K, followed by an appropriate fluorescently labeled polyclonal anti-IgG secondary antibody. The obtained fluorescence signal patterns revealed that, albeit with some differences in the fluorescence AU values, both 12G4 and 3C23K recognized only the two peptides (P5 and P6) that contain the DRAQVEM sequence (Table 1). This demonstrates that the humanization and affinity maturation processes did not modify the epitope recognized by 3C23K compared with the parental mouse antibody 12G4.

**Table 1:** Epitope mapping: mouse 12G4 *vs* human 3C23K

Position AA			12G4 binding (AU fluorescence)	3C23K binding (AU fluorescence)
Peptide 1	1–20	PPNRRTCVFFEAPGVRGSTK	< 1000	< 1000
Peptide 2	11–30	EAPGVRGSTKTLGELLDTGT	< 1000	< 1000
Peptide 3	21–40	TLGELLDTGTELPRAIRCLY	< 1000	< 1000
Peptide 4	31–50	ELPRAIRCLYSRCCFGIWNL	< 1000	< 1000
Peptide 5	41–60	SRCCFGIWNLTQDRAQVEMQ	37300 ± 5000	28400 ± 5000
Peptide 6	51–70	TQDRAQVEMQGCRDSDEPGC	24500 ± 4300	26000 ± 5100
Peptide 7	61–80	GCRDSDEPGCESLHCDPSPR	< 1000	< 1000
Peptide 8	71–90	ESLHCDPSPRAHPSPGSTLF	< 1000	< 1000
Peptide 9	81–100	AHPSPGSTLFTCSCGTDFCN	< 1000	< 1000
Peptide 10	91–110	TCSCGTDFCNANYSHLPPPG	< 1000	< 1000
Peptide 11	101–120	ANYSHLPPPGSPGTPGSQGP	< 1000	< 1000
Peptide 12	111–130	SPGTPGSQGPQAAPGESIWM	< 1000	< 1000
Peptide 13	113–132	GTPGSQGPQAAPGESIWMAL	< 1000	< 1000
Control	47	WNLTQDRAQVEMQGCRDSDE	11200 ± 2000	16600 ± 2300

Then, the potential cross-reactivity of 3C23K with other human proteins that display homology with the DRAQVEM sequence was evaluated. BLAST analysis identified 13 human proteins harboring homologous sequences (Table 2). Peptides of 20 amino acids containing these sequences were synthetized and immobilized on glass slides, as before. 3C23K bound weakly to only three of them (Table 2). Indeed, the fluorescence AU values obtained for IEEAFARAQVEMKAVHENLA from the kinesin-like protein KIFC3, for ARLELERAQLEMQSQQLRES from the brain leucine zipper (BRLZ) protein and for QLDFFDRAQIEQVIANCEHK from the Nup205 nucleoporin were 6%, 22% and 10%, respectively, of the value obtained with the MISRII DRAQVEM peptide. Moreover, these three proteins are localized in the cytoplasm or in the nucleus and therefore, should not be accessible to the MAb 3C23K *in vivo*.

**Table 2:** Binding of 3C23K to 13 human sequences homologous to the 7AA epitope sequence DRAQVEM identified by BLAST

Protein*	Peptide sequence**	3C23K binding (AU fluorescence)
MISRII (Q16671)	WNLTQ**D**R**A**QVEMQGCRDSDE	5200 ± 600
kinesin-like protein KIFC3 (AAH47051/Q9BVG8)	IEEAFAR**A**QVEMKAVHENLA	310 ± 80
BRLZ, brain leucine zipper protein (AAR83719/A6NC98)	ARLELER**A**QLEMQSQQLRES	1100 ± 100
unnamed protein product (BAG62992/B4DWD0)	NQLKDAI**A**QVEMDLKRLRDP	< 200
integrin alpha D, CD11d (AAB60630/Q13349)	GQEAFMR**A**QMEMVLEEDEVY	< 200
tektin 2 (EAX07391/Q9UIF3)	SRFNK**D**R**A**EAEMKAATELRE	< 200
DNA polymerase iota chain A (3H40_A/Q9UNA4)	VDLDCFY**A**QVEMISNPELKD	< 200
Nup205 (nucleoporin) (AAH44255/Q92621)	QLDFF**D**R**A**QIEQVIANCEHK	520 ± 20
integrin alpha D precursor (NP_005344/Q59H14)	GQEAFMR**A**QMEMVLEEDEVY	< 200
titin, isoform N2-B (NP_003310/Q8WZ42)	EGILT**D**R**A**QIEVTSSFTMLV	< 200
PITPNB (phosphatidylinositol transfer protein) (CAQ68294/B3KYB6)	HIDIA**D**RSQVEPADYKADED	< 200
LYPDC1 (AAH40046/Q8IXM0)	QERVD**D**R**A**EVEKRLREGEED	< 200
TMEM64 (transmembrane protein 64) (AAI13829/Q6YI46)	MFYVVHR**A**QVELNAAIVACE	< 200
PMS2 (mismatch repair endonuclease) (BAD89425/P54278)	PSDPT**D**R**A**EVEKDSGHGSTS	< 200

* Genbank and Uniprot references are indicated in parenthesis.

** Residues known to be critical for 12G4 binding (25) are in bold and identical residues found in the same position in the DRAQVEM sequence of MISR-II are underlined.

### Structural comparison of the CDRs of ch12G4, h12G4 and 3C23K

The humanization and maturation processes resulted in several variants with modifications in the light and heavy chain sequences and with different affinities for MISRII. The same homology building approach was used to generate 3D models of the antibody before and after humanization and before and after affinity maturation (Figure 1D). Superimposition of the ch12G4 and h12G4 models showed that, despite differences in the primary sequence, the spatial conformation of the main chain atoms was very similar in both antibodies, as indicated by the low rmsd value of 1.179 Å (Figure 1D). Superimposition of h12G4 and 3C23K also showed that the four mutations generated during the affinity maturation process (I54T, S56P, E68K and Q3R) had a very small effect on the antibody topology, as indicated by the very low rmsd value (0.126 Å) (Figure 1D). Taken together, these results suggest that the binding affinity variations observed during 12G4 humanization were not related to major changes in CDR spatial conformation. Nevertheless, it is worth to note that the three VL mutations are located inside (S56P) or surrounding (E68K, I54T) the CDR-L2 hypervariable loop. Although our molecular model did not reveal any major conformational change, it is not possible to exclude that these mutations, particularly S56P and I54T in the Vernier zone, affected CDR-L2 conformation or flexibility upon interaction with MISIIR. Moreover, CDR-L2 is close to CDR-H3 that contains two arginine residues (R106 and R114) critical for the binding to MISRII. Indeed, their substitution by alanine induced a drastic loss of binding affinity (as determined by alanine scanning experiments, data not shown). Therefore, the mutations in the CDR-L2 region could indirectly affect the antibody affinity by positioning the side-chain of the CDR-H3 arginine residues for optimal interaction (Supplementary Figure 1D).

Then, the effect of these mutations on the antibody electrostatic surface potential was calculated with the Delphi program. The humanization of 12G4 introduced a highly negative patch (red) in a highly positive region (blue), as a consequence of the A68E mutation (Figure 1E). During the affinity maturation, the glutamic acid residue was then replaced by a lysine residue (K68), restoring the initial charge in this region and contributing to the increase in binding affinity.

### 3C23K production in YB2/0, CHO or HEK293 cells, glycosylation analysis and effect on binding to Fcγ receptors

Oligosaccharide analysis of 3C23K expressed in YB2/0 (EMABling^®^ version; 3C23K) [30], CHO-S (3C23K-CHO) or HEK293 (3C23K-HEK293) cells (used as comparators for functional assays) revealed two clearly different glycosylation patterns (Supplementary Figure 1E). The percentages of fucosylated, galactosylated and bisecting GlcNAc isoforms were 33.0%, 57.2% and 1.8% for 3C23K and 94.6%, 54.4% and 2.0%, for 3C23K-CHO, respectively. The effect of these glycosylation differences on the binding to Fc©Rs was analyzed by SPR. Binding affinity for hFcγRIIIa and hF cγRIIIb was clearly increased following fucose reduction (1–12 nM and 86.0 nM for 3C23K compared with 31–164 nM and 378 nM for 3C23K-HEK293, respectively), but not for the other FcγRs (hFc©RI, hFcγRIIa, hFc©RIIb) (Table 3).

**Table 3:** Affinity constants (K_D_) of low-fucosylated 3C23K and glycosylated 3C23K-HEK293 for the different human Fcγ receptors

	Antibody	
Receptor	3C23K	3C23K-HEK293
Fc©RI/CD64	0.1–2.7*	0.2–3.6*
Fc©RIIa/CD32a	176	182
Fc©RIIbc/CD32bc	316	378
Fc©RIIIa/CD16a	1–12*	31–164*
Fc©RIIIb/CD16b	86	378

Affinity constants are expressed as K_D_ in nM. *K_D1_ and K_D2_ were calculated by using heterogeneous fitting model.

The K_D_ values, expressed in nM, were calculated by using a steady-state fitting model, but for FcγRI and FcγRIIIa. FcγRI and FcγRIIIa do not adhere to the one-to-one binding model and fit well using a heterogeneous ligand model (60). The fitting curves and data are given in supplementary data. The K_D_ values of 3C23K and 3C23K-HEK293 were obtained with the same fitting model, thus allowing comparing the data for each receptor, although the precise binding mechanism has not been elucidated yet and the affinity values reported can vary (60).

### *In vivo*, ^177^Lu-3C23K preferentially accumulates in ovarian tumor cell xenografts that express MISRII

**Figure 2:**
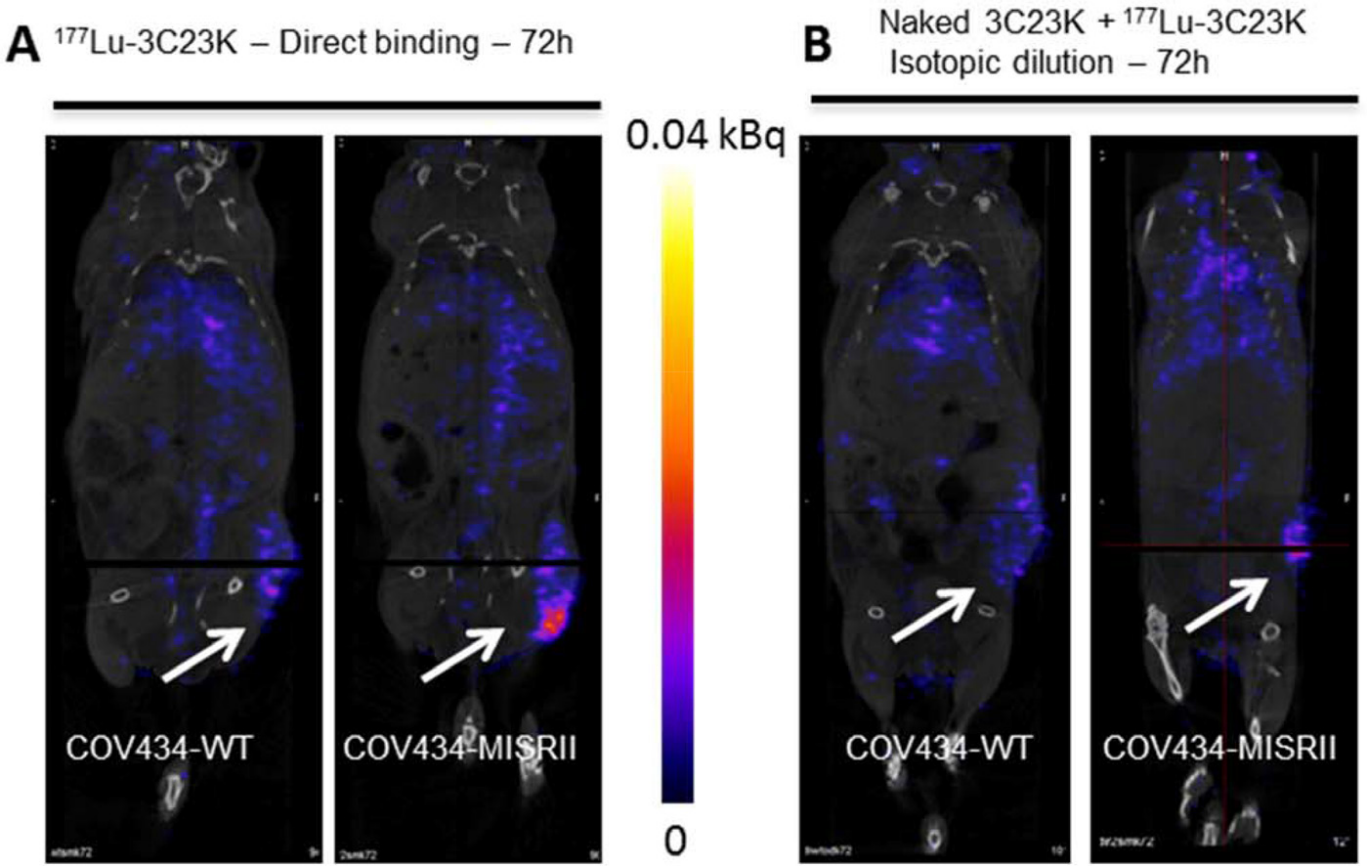
SPECT/CT imaging of COV434-MISRII and COV434-WT cell-derived xenografts in nude mice Representative images obtained (**A**) at 72 hours post-i.p. injection of ^177^Lu-3C23K3K (direct binding), (**B**) at 72 hours post-injection of ^177^Lu-3C23K that was administered 10 minutes after injection of naked 3C23K (20 mg/kg) (isotopic dilution).

A SPECT/CT-based study was designed to evaluate the *in vivo* biodistribution of the 3C23K MAb in nude mice xenografted with COV434-MISRII or COV434-WT (very low MISRII expression) cells. For this purpose, 3C23K was radiolabeled with Lutetium 177, a radionuclide that could be used for diagnostic purposes. Representative SPECT/CT images at 72 hours post-injection (Figure 2A) clearly showed that ^177^Lu-3C23K specifically localized in COV434-MISRII, but not in COV434-WT cell-derived tumor xenografts. To confirm the specificity of 3C23K binding *in vivo*, an isotope dilution experiment was carried out in which a large molar excess of non-radiolabeled 3C23K (20 mg/kg; therapeutic dose) was injected 10 minutes before injection of ^177^Lu-3C23K. Pre-injection of unlabeled MAb 3C23K decreased ^177^Lu-3C23K uptake in COV434-MISRII tumors to a level similar to that observed in COV434-WT xenografts (Figure 2B).

### 3C23K anti-tumor efficacy *in vivo*

We previously verified in mice xenografted with COV34-MISRII cells (OC model) that: (i) 3C23K anti-tumor efficacy was not significantly different when using different treatment schedules (2 or 3 injections of 10 mg/kg 3C23K/week for 4 or 6 weeks) (Supplementary Figure 2A) and (ii) the anti-tumor activity of 3C23K administered intraperitoneally (i.p.) or intravenously (10 mg/kg, Q3-4D12) was almost similar (Supplementary Figure 2B). Based on these data, the following three experiments were designed with the simplest protocol (i.e., 2 i.p. injections per week for 4 or 6 weeks).

### 3C23K reduces COV434-MISRII tumor growth more efficiently than 12G4

**Figure 3:**
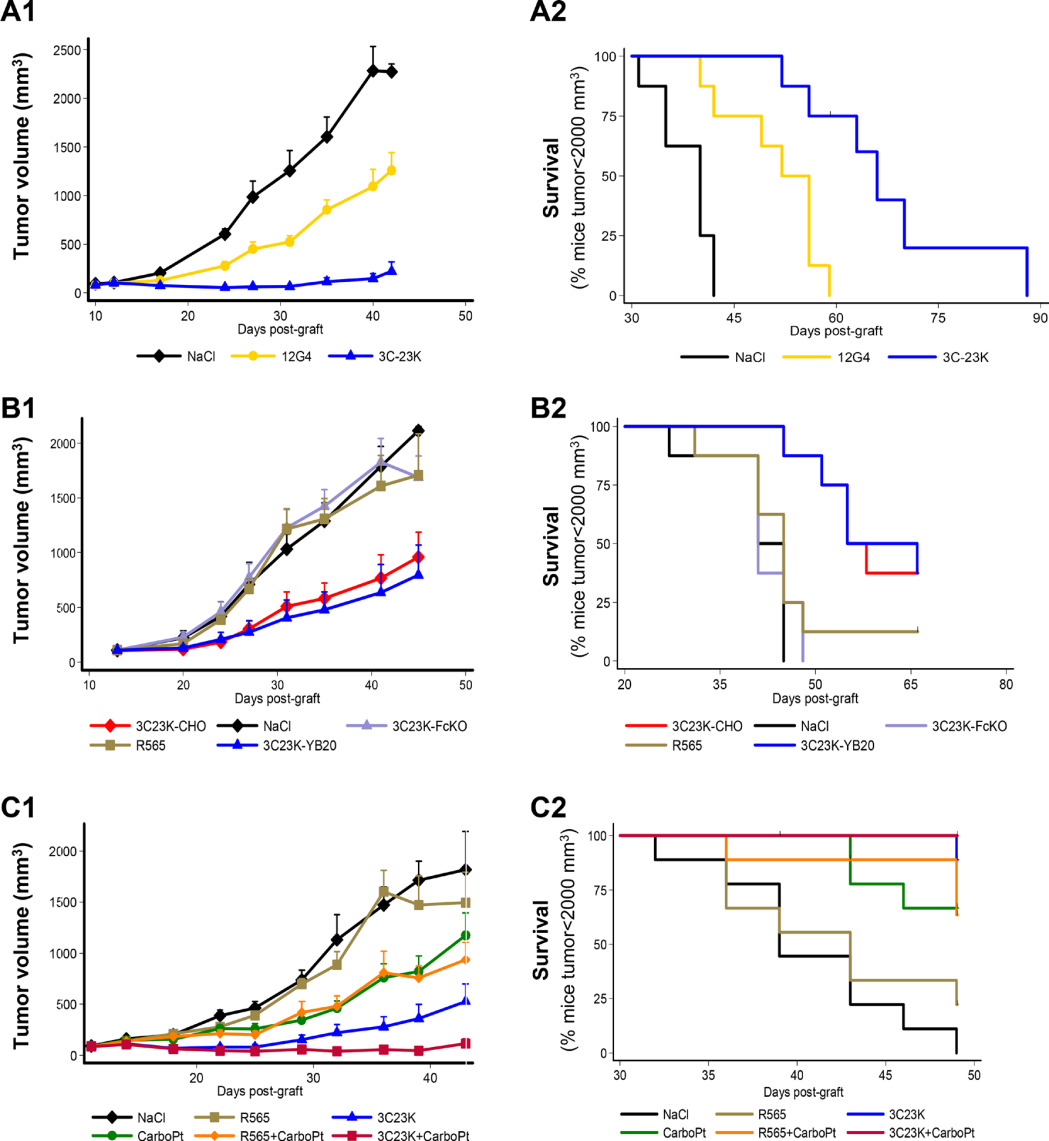
*In vivo* efficacy of EMABling^®^ 3C23K (low-fucose form) in mice xenografted with COV434- MISRII human ovarian cancer cells (**A**) Comparison with murine 12G4 or NaCl, as vehicle (7–8 mice/group). (**B**) Comparison with 3C23K-CHO (normal fucose form), 3C23K mutated in the Fc domain (no binding to Fc receptors) and the irrelevant R565 antibody (9 mice/group) and (**C**) Association with 60 mg/kg carboplatin (CarboPt). Results are presented as (1) tumor growth curves (mean and 95% CI upper bound) and (2) Kaplan–Meier survival curves (percentage of mice with a tumor volume lower than 2,000 mm^3^ as a function of time after graft).

To compare 3C23K and 12G4 efficacy, 12G4, 3C23K or vehicle (NaCl) were administered i.p. in mice with established COV434-MISRII cell-derived tumors (7–8 mice/group) twice a week for 6 weeks (18 injections in total) at about 10 mg/kg/injection, Q2-3D18. Both 12G4 and 3C23K significantly inhibited tumor growth compared with vehicle (*p* = 0.0012 and *p* = 0.0001, respectively) (Figure 3A1). However, 3C23K displayed a stronger anti-tumor activity than 12G4, as the tumor volumes in this group were significantly smaller than in the 12G4 group at all times points (*p* < 0.001). In this model and at this dose, 12G4 displayed only a partial anti-tumor activity, compared with vehicle. Indeed, the Kaplan-Meier survival curves highlighted a significant difference in survival between the 12G4- and 3C23K-treated groups (*p* = 0.0024) (Figure 3A2). This could be attributed to a better efficiency of human IgG1 than murine IgG1 in our mouse model (human cell-derived tumors in *nude* mice) due to the lower affinity of FcγRIIIa for mouse IgG1, as already reported by Bruhns *et al*. [34]. Moreover, Overdijk et al [35] demonstrated that human IgG1 and IgG2 activate murine polymorphonuclear leukocytes (PMNs) more potently than mouse IgG1.

### Mutated 3C23K that cannot bind to Fc receptors has no effect on COV434-MISRII tumor growth

A second study was designed to evaluate and compare in mice bearing COV434-MISRII tumors the efficacy of 3C23K (low fucose on the Fc fragment), 3C23K-CHO (high-fucose form) and of 3C23K with a mutation in the Fc domain (3C23K-FcKO; no binding to Fc receptors) (9 animals/group; 10mg/kg, one i.p. injection/week for 4 weeks, Q7D4). 3C23K and 3C23K-CHO significantly reduced tumor growth compared with 3C23K-FcKO (respectively, *p* = 0.023 and *p* < 0.0001) or vehicle (NaCl) (respectively, *p* = 0.012 and *p* < 0.0001) (Figure 3B1). Similarly, survival was significantly higher in mice treated with 3C23K and 3C23K-CHO than with 3C23K-FcKO or vehicle (*p* < 0.0001) (Figure 3B2). As already described by different authors for other MAbs, nude mice are not a good model to highlight differences in anti-tumor efficacy between antibodies with different levels of fucosylation [36]. Such difference might only be observed using human FcγRIII transgenic mice, as demonstrated by Junttilia et al. for the anti-HER2 antibody trastuzumab [36].

### 3C23K anti-tumor effect is enhanced when combined with carboplatin

A third *in vivo* study was performed to evaluate the anti-tumor activity of 3C23K (10 mg/kg, Q3-4D12) and carboplatin (60mg/kg Q7D4; a suboptimal and non-toxic dose) as monotherapies or in association in female nude mice xenografted with COV434-MISRII cells. Vehicle (NaCl) and the irrelevant antibody R565 (10 mg/kg, Q3-4D12), alone or in association with carboplatin, were used as controls (9 animals/group). 3C23K alone and the 3C23K + carboplatin combination had the highest inhibitory effect on tumor growth over time, compared with NaCl (*p* < 0.001) (Figure 3C1). At the end of the experiment, only 23 of the 54 analyzed mice had a tumor volume greater than 2000 mm^3^. Survival was significant longer in the 3C23K group than in the vehicle and R565 groups (*p* < 0.001). No difference was observed between treatment with vehicle and R565 (*p* = 0.796) (Figure 3C2). In these conditions, 3C23K alone displayed a significantly greater anti-tumor activity than carboplatin alone (*p* < 0.001), and the carboplatin + 3C23K combination displayed a stronger anti-tumor activity (*p* < 0.001) than each compound alone compared with vehicle (Figure 3C2). The association carboplatin + 3C23K showed an additive effect in this *in vivo* experiment. Treatments did not significantly affect body weight and could not been considered as toxic.

### Analysis of 3C23K mechanisms of action

### Analysis of COV434-MISRII cell responsiveness to MIS

**Figure 4:**
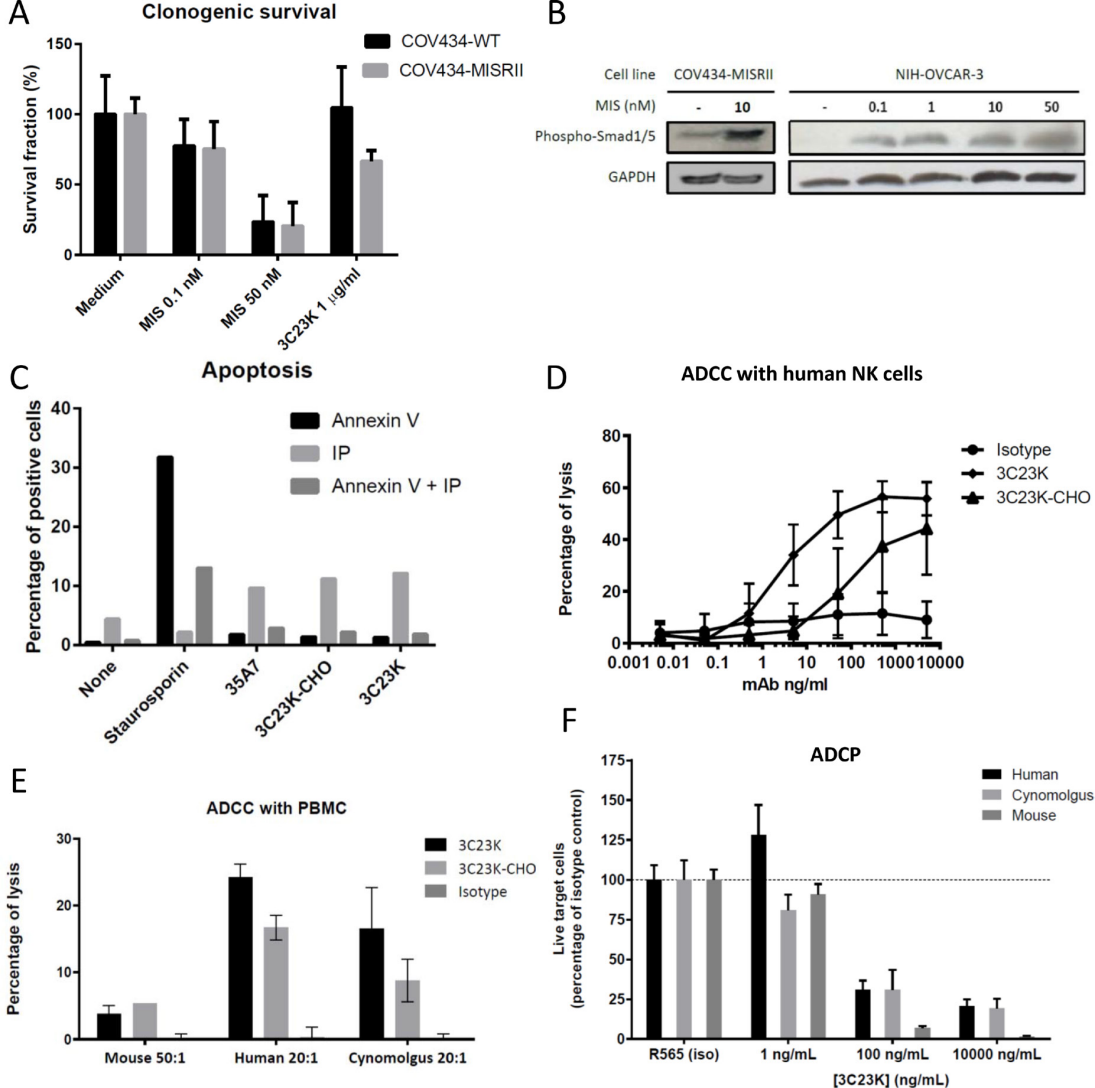
*In vitro* studies to determine 3C23K mechanisms of action (**A**) Clonogenic assay: Clonogenic survival of COV434-WT and COV434-MISRII cells incubated or not (medium) with MIS (0.1 or 50 nM) or 3C23K (1 or 100 μg/ml). After 15 days of culture in the presence of MIS or 3C23K, colonies were fixed with a methanol/acetic acid solution (3:1), stained with 10% Giemsa and counted. (**B**) Smad1/5 phosphorylation: Western blot analysis of serum-deprived (for 48 hours) COV434-MISRII (left panel) or NIH-OVCAR-3 (right panel) cell extracts obtained after incubation or not with MIS l (0.1, 1, 10 or 50 nM) for 1 hour using anti-phospho-Smad1/5 (Ser463/465) (41D10) rabbit MAb. MIS induced Smad1/5 phosphorylation in MISRII-overexpressing COV434 and NIH-OVCAR3 cells at all the tested concentrations in a dose-dependent manner. (**C**) Apoptosis: After incubation with 50μg/ml MAb (as indicated) or 150 nM staurosporin (positive control) for 24 hours, COV434-MISRII cells were stained using the Annexin V-FITC Apoptosis Detection Kit (Beckman Coulter IM3614). Results of one representative experiment out of four are shown and are expressed as the percentage of cell labeled with Annexin V, propidium iodide (PI) or both. (**D** and **E**) ADCC: COV434-MISRII cells were incubated with human NK cells (E:T ratio = 10) purified from healthy donors’ peripheral blood (D) or human, cynomolgus monkey or mouse PBMC (E:T ratio indicated in the figure) (E) and increasing concentrations of antibody (3C23K, 32C3K-CHO or irrelevant MAb) at 37°C for 4 hours. The lysis of target cells was assessed by quantifying the release of lactate dehydrogenase (LDH) by target cells in the supernatant and calculated according to the formula: % lysis = [(ER-SR)/(100-SR)]-[(NC-SR)/(100-SR)], where ER, SR and NC represent the experimental LDH release, the spontaneous LDH release (target cells without NK cells and without antibody) and the natural cytotoxicity (target cells + NK cells without antibody), respectively. (**F**) ADPC: COV434-MISRII target cells labeled with the CMFDA dye (CellTracker^™^Green, Life Technologies) and pre-incubated with increasing concentrations of antibody (3C23K or irrelevant MAb) at room temperature for 30 minutes were mixed (10:1 E:T ratio) with macrophages derived from monocytes obtained from human or cynomolgus monkey PBMC or mouse bone marrow. Living target cells were quantified by flow cytometry after 3 (human and cynomolgus monkey macrophages) or 5 days (mouse macrophages). Results are expressed as percentages relative to the corresponding isotype control.

Before evaluating the possible mechanisms of action of the MAb 3C23K, clonogenic assays were performed to determine whether the COV434-MISRII cell line is sensitive to MIS, the natural ligand of MISRII, and, thus, to confirm that it is a relevant model. The parental COV434-WT cell line was used as control. MIS reduced the clonogenic survival of both cell lines (Figure 4A). Specifically, the number of clones was decreased by about 25% with only 0.1 nM MIS and up to 80% with 50 nM MIS. These results compared favorably with those obtained by Masiakos et al. who used 100 nM MIS and different OC cell lines [16]. They also confirmed that both COV434 cell lines contain the machinery required for MIS/MIS receptor signaling and that they respond to MIS even at very low levels of MISRII expression (almost undetectable by FACS in COV434-WT cells [20]). On the other hand, 1 μg/ml 3C23K decreased clonogenic survival only in COV434-MISRII cells by about 35% compared with untreated cells (Figure 4A), demonstrating that 3C23K needs a sufficient MISRII expression level for its activity (the antigenic density in COV434-MISRII cells was 2 × 10^4^ receptors/cell by using Qifikit [20]).

To further confirm that the MIS pathway was functional and potentially activated in COV434-MISRII cells, the level of phosphorylated Smad-1/5 was evaluated following incubation with MIS. Indeed, MIS binds to MISRII that in turn, recruits and interacts with one of at least three candidate type I receptors (ALK2, ALK3 and ALK6) [12]. Subsequently, the relevant type I receptor mediates downstream signaling by phosphorylating Smad-1/5/8 proteins that then binds to the Smad4 coactivator to form one unit that translocates into the nucleus to induce growth inhibition [12]. In COV434-MISRII cells, MIS-dependent Smad signaling could be induced like in NIH-OVCAR3 cells that express endogenous MISRII (Figure 4B).

### 3C23K does not induce apoptosis of COV434-MISRII cells

The finding that 3C23K-FcKO did not have any *in vivo* effect (Figure 3B) suggested that 3C23K binding to MISRII might not induce a direct anti-proliferative effect in the absence of immune effector cells. Indeed, incubation of COV434-MISRII cells with 50 μg/ml of 3C23K or 3C23K-CHO for 24 hours did not induce any significant increase in apoptosis or necrosis compared with an irrelevant antibody (anti-CEA MAb 35A7) (Figure 4C), differently from what reported for MIS [18, 32] and for the mouse 12G4 MAb [20]. This difference with 12G4 could be explained by the different amino acid sequence specifically in the hinge region, as recently reported for rituximab by Könitzer *et al*. [37].

### 3C23K induces ADCC and ADCP, but no CDC

Based on the findings that 3C23K-FcKO did not have any *in vivo* effect (Figure 3B) and that 3C23K did not induce apoptosis *in vitro* (Figure 4B), the potential 3C23K Fc-related mechanisms of action were investigated. First, CDC was assessed using a cytotoxicity detection kit based on the measurement of lactate dehydrogenase (LDH) activity released by damaged cells. COV434-MISRII cells were incubated with increasing concentrations of 3C23K or 3C23K-CHO (0 to 5,000ng/ml) in the presence of baby rabbit serum, as a source of complement (1/10 dilution). After 1 hour of incubation at 37°C, no cell lysis was observed, whatever the treatment (data not shown). Lack of CDC activity in our experimental conditions could be attributed to the too low MISRII antigenic density (2x10^4^ receptors/cell [20]) for CDC. Moreover, FACS analysis of the expression of membrane-bound complement regulatory proteins (mCRPs) [38] showed no expression of CD55, but clear expression of CD46 and CD59 (MFI = 6.9 and 16.0, respectively).

ADCC was first assessed using human NK cells, as standard ADCC effectors cells. 3C23K and 3C23K-CHO induced ADCC of COV434-MISRII cells (Figure 4D). The maximal lysis obtained at the highest antibody concentration (5000 ng/ml) was 56% with 3C23K and 46% with 3C23K-CHO; 50% of the maximal lysis was obtained with 133.35 ng/ml of 3C23K-CHO and with 2.9 ng/ml of 3C23K (a 46-fold lower concentration).

To perform experiments closer to the *in vivo* situation, ADCC was then measured using human, cynomolgus monkey and mouse peripheral blood mononuclear cells (PBMC). Human PBMC purified from three different donors heterozygous for the FcγRIIIa-158 polymorphism induced a clear ADCC at a 20:1 effector to target cells (E:T) ratio and a MAb concentration of 1000 ng/ml. 3C23K was more efficient with up to 24% specific lysis compared with 17% for 3C23K-CHO (Figure 4E). Depending on the experiment, 50% of maximal lysis was obtained with 2.5- to 4.5-fold lower concentrations of 3C23K than 3C23K-CHO. Very similar results were obtained with cynomolgus monkey PBMC, but with lower maximum lysis levels (17% and 9% for 3C23K and 3C23K-CHO, respectively) (Figure 4E). ADCC was more difficult to observe using murine PBMC because only one experiment in three gave some results with an E:T ratio of 50 and a MAb concentration of 30 μg/ml (Figure 4E). With murine PBMC, 3C23K did not show any advantage relative to 3C23K-CHO.

The low ADCC level obtained with murine PBMC and the similarity of ADCC level between 3C23K and 3C23K-CHO were not in agreement with our *in vivo* results in mice, suggesting that ADCC might not be the main mechanism of action. ADCP was thus investigated, as an alternative mechanism of action for 3C23K. COV434-MISRII cells labeled with the cell tracker CMFDA were incubated with 3C23K or an irrelevant antibody (R565) for 30 minutes and then added to human, cynomolgus monkey or murine macrophages for 5, 5 or 3 days, respectively. Afterwards, the number of living COV434-MISRII cells, considered as a representative and easy parameter for assessing ADCP efficacy and for comparison with ADCC, was measured by FACS and expressed as a percentage relative to control (irrelevant antibody) (Figure 4F). In this assay, murine macrophages were even more efficient than human and cynomolgus monkey macrophages with only 10% of live target cells left after incubation with 100 ng/ml 3C23K compared with about 25% of live cells when using human or cynomolgus monkey macrophages (Figure 4F).

## DISCUSSION

OC has an overall cure rate of less than 40% and, except for a moderate effect observed with bevacizumab, no significant treatment progress has been made so far. New therapies are, therefore, required to improve OC outcome. Targeted therapies using MAbs have substantially improved the management of many solid tumors and hematologic malignancies [39] and may also be effective in OC. The emergence of new targets, such as sperm protein 17 [40] and MISRII [25], may offer new hope.

In this study, we describe the development and testing of a humanized and glyco-engineered version of the anti-human MISRII mouse MAb 12G4 [25] in order to reduce its immunogenicity and to increase its effector activity, in view of its transfer to the clinic. Using the classical humanization technique based on the transfer of mouse CDRs to the human FRs followed by the MutaGen^™^ technology [27, 33] and combining mutations from different variants, we isolated the 3C23K MAb that exhibits the same epitope DRAQVEM and a higher antigen affinity than the parental 12G4 antibody.

Then, 3C23K was expressed in YB2/0 cells using the EMABling^®^ technology to increase the antibody interaction with the low/medium affinity FcγRIIIa receptor that is mainly expressed on NK cells and macrophages [30]. This property is related to the lower expression of the *Fut8* gene in rat myeloma YB2/0 cells compared with other commonly used cell lines, such as CHO cells [30]. Two EMABling^®^ MAbs, the anti-RhD roledumab [41] and the anti-CD20 ublituximab [42], are currently in clinical development. Moreover, we [29, 41] and other groups [43–45] have demonstrated that such improved effector functions are associated with higher therapeutic efficacy in humans, especially in oncology.

As expected, 3C23K-YB2/0 displayed higher binding affinity for FcγRIIIa receptor than high-fucose content 3C23K (Table 3). This enhanced binding to FcγRIIIa receptor gave to 3C23K a stronger cytotoxic activity, as measured by ADCC, which was 32-fold higher than that of the same 3C23K molecule produced in CHO or HEK293 cells (Figure 4C). One of the potential drawbacks of second generation therapeutic antibodies with enhanced Fc-related mechanisms of action is that their enhanced efficacy on tumor cells could perhaps result in higher toxicity if the target antigen is also expressed in normal tissues, even at low antigenic density. Thus, to assess MISRII expression in normal tissues, we analyzed by qPCR a series of 48 human tissue samples (Supplementary Figure 3). MISRII expression was observed essentially in ovary, which was used as calibrator (relative quantification, RQ = 1), and, to a lesser extent, in adrenal gland (RQ = 0.486), testis (RQ = 0.267), penis (RQ = 0.169) and pancreas (RQ = 0.138) (Supplementary Figure 3). This restricted expression profile of MISRII is, thus, well adapted to second generation therapeutic antibodies with enhanced Fc-related mechanisms of action, such as 3C23K.

3C23K-mediated ADCC could be considered as elevated, on the basis of MISRII antigenic density (about 2 × 10^4^ receptors per cell by Qifikit measurement [20]). However, a recent study on MISRII regulation showed that this receptor clusters at the cell surface [46]. In these clusters, MISRII density is very high and this might promote ADCC. This observation makes of MISRII-3C23K an ideal receptor-MAb couple for OC immunotherapy.

The strong anti-tumor efficacy of 3C23K was confirmed *in vivo*. Intraperitoneal administration of 3C23K significantly delayed the growth of COV434-MISRII tumor xenografts and its effect was additive with that of carboplatin (Figure 3A and 3C). The antibody anti-tumor cytotoxic effect was abolished when Fc-mutated 3C23K, which cannot bind to the Fc receptor, was used (Figure 3B).

Taken together, these data suggest that 3C23K binding to FcγRIII plays a major role in promoting ADCC. These results are in agreement with those by Liu *et al*. [47] using anti-CD20 (in hematologic cancers) or anti-HER2 (in solid tumors) therapeutic antibodies. Specifically, they found that the higher affinity of afucosylated antibodies for FcγRIIIa, compared with high-fucose antibodies, stimulates the activation of signaling molecules that potentiate NK cell cytotoxic activity, thus increasing ADCC efficiency [47, 48].

Our *in vivo* data also highlight a difference between the efficacy of the murine 12G4 MAb and the humanized 3C23K MAb (Figure 3A). This could be attributed to the major differences between human and mouse FcR systems for specific IgG subclasses. It has been shown that differently from other mouse FcγRs, mouse FcγRIV has a distinct IgG subclass specificity with no affinity for mouse IgG1 or IgG3 and high-affinity for mouse IgG2a and IgG2b. Conversely, FcγRIII is a low-affinity receptor for mouse IgG1, IgG2a and IgG2b (for review [34]). In parallel, it was clearly demonstrated using a panel of anti-EGFR MAbs and A431 cells that human IgG1 are the most potent in inducing mouse PMN-mediated ADCC compared with murine IgG1 or IgG2 [35].

ADCC is considered the main immune-related mechanism of action of therapeutic MAbs, as reported at the preclinical [49] and clinical levels [50, 51] for different anti-carcinoma antibodies, including cetuximab and trastuzumab. After confirmation of the high ADCC potential of 3C23K with human NK cells (50% maximal lysis obtained with 2.9 ng/ml of 3C23K compared with 133.35 ng/ml of 3C23K-CHO; Figure 4), we investigated its ADCC activity with PBMC. 3C23K induced high ADCC with human and cynomolgus monkey PBMC and very limited ADCC with murine PBMC (Figure 4). As this was not in agreement with our *in vivo* results, we investigated ADCP and found that it was very high with 3C23K and murine macrophages. This can be explained by the fact that human IgG1 exhibit the strongest binding to murine FcγRIV expressed on murine macrophages [52] and ADCP certainly contributes to 3C23K *in vivo* efficacy and might compensate at least partially the lack of activation of murine NK cells (Figure 3). Overall, these data strongly suggest that *in vivo* pre-clinical experiments underestimate the possible 3C23K efficacy in the clinic.

Besides efficacy, toxicity is an important issue in the development of a new drug. As 3C23K does not bind to mouse MISRII, our experiments in mice gave us no clue on its possible toxicity towards normal tissues. However, as a first step towards clinical trials, we performed regulatory toxicological studies in cynomolgus monkeys and 3C23K did not elicit any sign of toxicity with no observed adverse effect level (NOAEL) at doses ≥ 300 mg/kg [53].

In conclusion, on the basis of the previous characterization of the mouse 12G4 MAb [20, 25] and the present data, the anti-MISRII MAb 3C23K appears to be a promising new EMABling^®^ therapeutic candidate for OC treatment. In 2016, a phase I clinical trial (NCT02978755) was initiated with 3C23K (now named GM-102) in gynecological cancers.

## MATERIALS AND METHODS

### Humanization of the murine MAb 12G4

12G4 variable regions were amplified from total RNA by using the 5′ RACE kit (Invitrogen). The variable regions of both chains were cloned into the TOPO TA cloning vector (Invitrogen) and were confirmed by sequencing (MWG). The human acceptor FRs were identified according to Pelat [54]. Briefly, the mouse sequences of the heavy and light chain Fv fragments were used for alignment to human germline sequences in the IMGT database (www.imgt.org). Based on these alignments, amino acid differences at specific positions between the human and murine FRs were detected. The properties of these residues (involvement in disulfide bridges or CDR flanking regions) were analyzed manually with the help of a “collier de perles” representation [31] and led to the construction of FRs for grafting the 12G4 CDRs of the heavy and light chains.

### Affinity maturation of humanized 12G4 (h12G4)

Based on the variable regions determined previously, 12G4 and h12G4 were reformatted as single chain Fv (scFv). The VH and VL domains were assembled by overlap PCR with an appropriate linker sequence [(GGGGS)3] and inserted in the pMG72 vector (Millegen). The humanized version of the scFv anti-MISRII antibody was used as template to build four libraries with MutaGen^™^ following the previously described procedure [27] and using different experimental conditions and human polymerases. The four libraries were mixed and rescued using the helper phage M13K07 (New England Biolabs). After overnight expression, phage-scFv particles were purified by PEG precipitation and titrated by infecting XL1-Blue cells (Stratagene).

The recombinant MISRII-Fc protein was produced using the FreeStyle^™^ MAX CHO Expression System (Invitrogen) and purified on protein A. For phage library panning, recombinant MISRII-Fc was immobilized onto Maxisorb (Nunc) at two concentrations: 1 μg (rounds 1–4) and 0.5 μg (rounds 5–6) per well. An aliquot of the scFv-phage particles in PBST/5% skimmed milk was added to the coated wells that were pre-blocked with 5% skimmed milk. After 2 hours of incubation at 37°C and several washes, bound scFv-phage particles were eluted by adding exponentially growing XL1-Blue bacteria. Infected XL1-Blue cells were then plated on Petri dishes and grown overnight, before being transferred to 2 × YT medium/glycerol and used for the next selection step.

After phage panning, the pairs of VL and VH domains of the selected variants (mouse and humanized anti-MSRII antibodies) were reformatted as Fab fragments. They were subcloned in the bicistronic bacterial expression vector pMG92 to introduce a V5 tag (Invitrogen) and a His6 tag, both at the C-terminus of the VH-CH1 chain. The Fab fragments were expressed overnight at 30°C in *E. coli* HB2151 cells by adding 0.5mM IPTG. The Fab fragments were then purified by one-step chromatography with Ni-NTA resin (Qiagen) and, after dialysis against PBS, were kept at 4°C until use.

### Binding characterization of the anti-MISRII antibodies

The binding of the different antibody clones to MISRII was evaluated by ELISA assay. The purified Fab fragments were evaluated with a similar ELISA assay at various concentrations (from 10 μg/ml to 156 ng/ml). Bound Fab fragments were detected by adding the HRP-labeled anti-V5 antibody (Invitrogen). The mouse (12G4) and humanized (h12G4) anti-MISRII antibodies in the IgG format were also compared in an ELISA assay using, respectively, anti-mouse IgG (HRP-conjugated) (Cell Signaling) and anti-human F(ab’)_2_ fragment (HRP-conjugated) (Interchim) antibodies.

Antigen recognition was also monitored by flow cytometry using 2 × 10^5^ COV434-MISRII cells. Cells were incubated with 100 μl of chimeric or humanized 12G4 at different concentrations (0 to 10μg/ml, final concentration) [20]. After washing, cells were incubated with 100μl (1:100) of a goat anti-human FITC-labeled antibody (Beckman Coulter). Cells were finally washed and analyzed with a flow cytometer (Beckman Coulter).

### Cloning of chimeric 12G4, humanized 12G4 and 3C23K

Chimeric 12G4 (ch12G4) was constructed and expressed as described previously [30]. Briefly, the VL and VH DNA sequences were subcloned sequentially into the polycistronic CHK622-08 vector that contains the promoter, Kozak sequence and the sequences of the human Kappa/IgG1 constant regions.

The DNA sequences coding for humanized 12G4 (h12G4) VL and VH were synthesized using Genscript and then cloned in CHK622-08 by digestion and ligation as described above, resulting in the HK622-18 vector. The DNA sequences coding for affinity-matured 3C23K VL and VH were obtained by directed mutagenesis of the phage clone 3C23 to introduce the VL E68K mutation. Signal peptides were added by PCR assembly using the humanized variable regions of h12G4 as template and then cloned in HK622-18, as described above. The resulting vector that expresses the humanized and affinity-matured 3C23K antibody was called HK622-18 MAO 3C23K.

### Production and purification of ch12G4, h12G4 and 3C23K

The different molecules were stably expressed, as previously described [30]. CHO-S, HEK293 or YB2/0 cells were stably transfected with the appropriate linearized expression vectors. Ch12G4, h12G4 and 3C23K antibodies were produced in YB2/0 cell using EMS (Invitrogen), 5% Ultra-low IgG fetal calf serum (FCS) (PAA) and 0.5g/l G418 for 5 to 7 days. 3C23K-CHO-S was produced in CHO-S cells using ProCHO4 (Lonza), 4mM glutamine and 1g/l G418 for 7 days.

MAbs were purified from culture supernatants by affinity chromatography using protein A sepharose (GE-Healthcare). The levels of aggregates and endotoxins were determined by gel filtration on Superdex HR/200 (GE-Healthcare) and by LAL testing, respectively. Antibody quality and purity were monitored by SDS-PAGE and Coomassie staining. In addition, the glycosylation patterns and core fucose percentage of each purified antibody were determined by high performance capillary electrophoresis laser induced fluorescence (HPCE-Lif) [55].

### Epitope mapping

Epitope mapping for 3C23K was performed by peptide array using the EpiFlag^®^ methodology (Innobiochips). Thirteen 20-amino acid peptides covering the 132 first C-terminal amino acids of MISRII were synthesized by solid phase peptide synthesis (Intavis AG). A peptide containing the epitope DRAQVEM in the central position was used as control.

Each peptide was characterized by RP-HPLC and MALDI-TOF MS and lyophilized. Peptide arrays were prepared by dissolving peptides at 0.1 mM in 0.01M PBS pH 7.4 and printing them on amine-modified glass slides (Arrayit). Microarrays were then saturated with PBS-M (PBS, 0.05% Tween20, 2.5% non-fat milk). After washes, slides were incubated with 20 μg/ml 3C23K in PBS-M at 4°C overnight. Bound antibodies were detected by adding a fluorescently labeled polyclonal anti-human IgG antibody (Abcam) at room temperature for 1 hour. Bound antibodies were detected using a TECAN LS Reloaded laser scanner (Tecan). Data were extracted and analyzed using the Array-Pro^®^ Analyzer software. The antibody binding potencies to the different peptides were compared using the Student *t*-test and the Statgraphics Centurion XV V15.2.06 (StatPoint, Inc) software. Differences were considered as statistically significant when *p* < 0.05.

### SPR analysis

SPR analyses were performed on a Bia3000 or T200 apparatus at 25°C in HBS-EP (GE Healthcare). For affinity measurements, MISRII was covalently immobilized (1000 RU) on a CM5 sensor chip using EDC/NHS activation, according to the manufacturer's instructions (GE Healthcare). Different concentrations (0.5–128 nM) of 12G4 or 3C23K were injected on immobilized receptor during 180 seconds. After 400 seconds of dissociation in running buffer, the sensor chip was regenerated using Gly-HCl pH 1.7. The K_D_ values, taking into account affinity and avidity, were calculated using a Langmuir 1:1 fitting model (BiaEvaluation3.2, GE Healthcare). Antibody-FcγR measurements were performed by single-cycle titration at 100 μl/min on FcγR (Sigma) captured on anti-His (R&D Systems) covalently immobilized at 4000–5000 RU level. The gamma receptor was injected at 20nM during 60 seconds and five increasing antibody concentrations (supplementary data) were injected (injection time = 60 seconds). After a dissociation step of 600 seconds in running buffer, sensor surfaces were regenerated using 5 μl of Glycine-HCl pH 1.7. Kinetic parameters were evaluated from the sensorgrams using a heterogeneous Ligand or steady-state fitting models on the T200evaluation software 3.0 (GE healthcare). All sensorgrams were corrected by subtracting the low signal from the control reference surface (without any immobilized protein) and buffer blank injections before fitting evaluation.

### Molecular modeling of anti-MISRII antibodies

All algorithms used for antibody modeling were extracted from the Discovery studio V3.1 software (Accelrys software Inc). The Fab structure of ch12G4 and h12G4 were modeled with a sequence homology approach using MODELER [56]. The best homologous templates were identified by BLAST search against a sequence database of known antibody 3D structures (extracted from the PDB database). The following PDB structures were the templates selected to build the initial model: i) 2OSL for the light and heavy chains of ch12G4; ii) 3EO9 and 2EH7 for the light and heavy chains of h12G4; and iii) 3E09 for the interface. CDR loops were specifically rebuilt and refined using the LOOPER algorithm.70. Starting from the h12G4 model, the 3C23K model was built by replacing the four mutated residues generated by the maturation affinity process: I47T, S49P, E54K in the VL and Q3R in the VH. The conformation of the mutated residues and of the surrounding residues that lie within a 5Å cutoff radius was optimized using the MODELER algorithm. The three models were superimposed based on their sequence alignment, and the main chain atom rmsd calculated using the 3DMA program. The electrostatic potential of the three antibodies was calculated using Delphi, a program that solves the Poisson-Boltzmann equation on a cubical lattice using the finite-difference technique [57].

### Cell lines and cell culture

The human GCT cell line COV434 [58], a kind gift from Peter I. Schrier (Department of Clinical Oncology, Leiden University Medical Center), was called COV434-WT in the present work. The COV434-MISRII cell line, a COV434-WT clone transfected with the cDNA encoding full-length human MISRII in the pCMV6 plasmid to stably express MISRII, was described by Kersual *et al*. [20]. Cells were grown in DMEM F12 medium containing 10% heat-inactivated fetal bovine serum (FBS), 0.1mg/ml streptomycin, 0.1IU/ml penicillin and 0.25mg/ml amphotericin B. COV434-MISRII cells were supplemented with 0.33 mg/ml geneticin. Cells were grown at 37°C in a humidified atmosphere with 5% CO_2_ and medium was replaced twice a week. Cells were harvested with 0.5 mg/ml trypsin/0.2mg/ml EDTA. All culture media and supplements were purchased from Life Technologies, Inc. (Gibco BRL).

### Antibodies

The murine anti-MISRII MAb 12G4 was described by Salhi et al. and Kersual et al. [20, 25]. Anti-idiotype factor VIII chimeric IgG1 R565 EMABling^®^ MAb and anti-CEA MAb 35A7 [20] were used as irrelevant antibodies.

### Normal tissues MISRII expression analysis by qPCR

For normal tissue MISRII expression studies, TissueScan Normal Tissues qPCR Arrays (HMRT102, OriGene Technologies) were probed for MISRII (Mix Hs01086650_g1 Applied Biosystems) and GAPDH (Mix 4310844e Applied Biosystems) expression in triplicate on a Real Time PCR thermal cycler (Model and Company). Cycle threshold (Ct) levels are inversely proportional to the amount of target nucleic acid in the sample. An ovary genomic sample was used as internal calibrator. MISRII relative quantification (RQ) was expressed as 2^−ΔΔCt^ where ΔΔCt = (ΔCt sample - ΔCt internal calibrator) and ΔCt = (Ct MISRII gene - Ct GAPDH endogenous control).

### Clonogenic survival

COV434-WT and COV434-MISRII cells were plated in 6-well/plates (200 cells per well) that had been pre-coated with poly-ornithine at 37°C for 2 hours. From the next day, cells were cultured in the presence of 0.1nM or 50nM MIS (Origene, USA) for 15 days. At day 15, colonies were fixed with a methanol/acetic acid solution (3:1), stained with 10% Giemsa and counted. Two independent clonogenic assays were performed. In the second experiment, COV434-WT and COV434-MISRII cells were plated at 400 and 200 cells per well, respectively.

### MIS pathway analysis

Cells were pre-treated by incubation in serum-free DMEM medium for 48 hours, followed by stimulation with MIS (0.1, 1, 10 or 50 nM) at 37°C for 1 hour. To obtain whole cell lysates, cells were harvested, washed twice with ice-cold PBS and lysed in lysis buffer (20 mM Tris-HCl, pH 7.5, 150 mM NaCl, 1mM Na_2_EDTA, 1mM EGTA, 1% Triton and complete protease inhibitor cocktail). Equal amounts of proteins (30 mg) were separated by 10% SDS-PAGE in non-reducing conditions. Proteins were transferred to PVDF membranes (Bio-Rad) by electroblotting. Membranes were blocked in 5% non-fat milk in TBS-T buffer and probed with the anti-phospho-Smad 1/5 MAb (1:1,000; Cell Signaling) at 4°C overnight. After washes, membranes were incubated with the anti-rabbit IgG HRP secondary antibody (1:10,000; Sigma) at room temperature for 1 hour. Proteins were visualized by enhanced chemiluminescence (ECL plus Western Blotting Detection System; Amersham Biosciences).

### Apoptosis assay

Approximately 350,000 cells were seeded in 6-well/plates and incubated or not with 50 μg/ml MAb, 20 nM MIS or 150 nM staurosporin, as positive control, for 24 h and then stained using the annexin V-FITC Apoptosis Detection Kit (Beckman Coulter IM3614). Both adherent and detached cells were collected and centrifuged at 1,000 rpm for 5 minutes. After washing with PBS, cells were stained with 130 μl of a mix containing 10 μl FITC-labeled annexin V and 20 μl propidium iodide (PI) in 100 μl annexin buffer on ice in the dark for 15 minutes. After addition of 400 μl annexin buffer, cells were analyzed by flow cytometry within 30 minutes and data analyses performed using the Kaluza Flow Analysis software (Beckman Coulter).

### ADCC assays

Human NK effectors cells from peripheral blood of healthy donors were purified by negative depletion (NK Cell Isolation Kit, Miltenyi Biotech). COV434-MISRII cells (30,000 cells/well of a 96-well flat bottom plate) were incubated with NK cells and increasing antibody concentrations (3C23K, 32C3K-CHO or irrelevant MAb) at 37°C for 4 hours. After incubation, supernatant was removed and lysis was measured by quantifying the amount of lactate dehydrogenase (LDH) released by target cells in the supernatant (Cytotoxicity Detection Kit LDH, Roche Diagnostic). The lysis percentage was calculated according to the formula: % lysis = [(ER-SR)/(100-SR)]-[(NC-SR)/(100-SR)] where ER, SR and NC represent the experimental LDH release, the spontaneous LDH release (target cells without NK cells and without antibody) and the natural cytotoxicity (target cells + NK cells without antibody), respectively.

PBMC used as effectors cells were of human, cynomolgus monkey and mouse origin. Human PBMC were from three different donors who were heterozygous for the *FCGR3A*-158 polymorphism. Blood samples were purchased from Etablissement Français du Sang (Nantes, France) and PBMC were isolated using a standard Ficoll procedure. Cynomolgus monkey PBMC were directly purchased from CytoxLab (Evreux, France) and were isolated from blood samples of individual cynomolgus monkeys using a standard Ficoll procedure. Human and cynomolgus monkey PBMC were frozen in FBS with 10% DMSO and stored in liquid nitrogen until use. Murine PBMC isolated from C57BL/6 mice were directly purchased frozen from AllCells (Alameda, CA, USA). ^51^Cr-labeled COV434-MISRII cells (1,500 cells/well) were incubated with PBMC and increasing antibody concentrations (3C23K, 32C3K-CHO or irrelevant MAb) at 37°C for 4 hours. Target cell lysis was measured by supernatant gamma counting. The lysis percentage was calculated according to the formula: % lysis = [(CPM_X_-CPM_spontaneous_)/(CMP_maximum_-CPM_spontaneous_] × 100 where X, spontaneous and maximum represent the experimental ^51^Cr release, the spontaneous ^51^Cr release (target cells without PBMC and without antibody) and the maximum ^51^Cr release (target cells alone in 0.75% triton X-100), respectively.

### ADCP assay

Monocytes were isolated from adult human or cynomolgus monkey blood samples purchased from Etablissement Français du Sang (Nantes, France) or CytoxLab (Evreux, France), respectively. PBMC were isolated using a standard Ficoll procedure and then plated in non-coated plates with RPMI alone at 37°C for 1 hour to allow the adhesion of monocytes to the plastic. Wells were then washed 3 times with 1ml RPMI/10% FBS to eliminate non-adherent cells. After 6 to 8 days of culture in RPMI with 10% FBS and 50 ng/ml human M-CSF (216-MC, R&D Systems, USA) (medium renewed at day 3–4), macrophages were detached by incubation on ice with ice-cold PBS containing EDTA (4 to 10 mM, depending on the adhesion strength) for 10–15 minutes, counted and used for ADCP assays. Frozen macrophages differentiated from monocytes isolated from the bone marrow of C57BL/6 mice were purchased from ScienceCell (San Diego, CA, USA). Macrophages were thawed following the supplier's instructions, resuspended in the specific macrophage culture medium (MaM, ScienceCell) and allowed recovering overnight before ADCP assay.

COV434-MISRII target cells (T) were extemporaneously labeled with the CMFDA dye (CellTracker^™^ Green, Life Technologies) following the manufacturer's instructions and pre-incubated with increasing concentrations of antibody (3C23K or irrelevant MAb) in RPMI/10% FBS at room temperature for 30 minutes before mixing with macrophages (effector cells: E) at a 10:1 E:T ratio. Alive target cells were detected by flow cytometry after 3 or 5 days incubation with human, cynomolgus monkey or mouse macrophages, respectively. Results were expressed as percentage of living cells relative to the isotype control.

### *In vivo* studies using OC xenografts

All animal experiments were performed in compliance with the guidelines of the French government and the regulations of the Institut National de la Santé et de la Recherche Médicale for experimental animal studies (agreement B34–172–27).

For all the *in vivo* experiments, female Swiss or athymic *nude* mice (6–8 week-old) (Harlan Laboratories, St Isle, France) were subcutaneously (s.c.) grafted on the right flank with 7.10^6^ human COV434-MISRII cells [20] in BD Matrigel (ratio 1:1) in a volume of 150μl at day 0 (D0). Mice were randomized when tumor volume was 60–150 mm^3^ at D12-D13 (*n* = 7–8 mice/group, for experiment A and *n* = 9 mice/group for experiments B and C). Treatments were all administered by intraperitoneal (i.p.) injection.

For experiment A, 3C23K, 12G4 or vehicle (NaCl) were injected i.p. twice a week for 6 weeks (18 injections in total) at about 10mg/kg/injection, Q2-3D18. For experiment B, 3C23K (anti-MISRII MAb produced in YB2/0 cells), 3C23K-CHO, 3C23K-FcKO, the irrelevant R565 MAb or vehicle (NaCl) were used. The treatment schedule was the same for all groups: 10 mg/kg in 200 μL, one i.p. injection/week for 4 weeks (Q7D4). In experiment C, 3C23K or the irrelevant antibody R565 was administered twice a week for 6 weeks (12 i.p. injections in total) at about 10mg/kg/injection, Q3-4D12. Carboplatin was administered once a week for 4 weeks (four i.p. injections in total) at the previously determined suboptimal dose of 60mg/kg/injection, Q7D4. Carboplatin was evaluated alone (Q7D4) or combined with 3C23K (carboplatin Q7D4 + 3C23K Q3-4D12) or with the irrelevant antibody R565 (carboplatin Q7D4 + R565 Q3-4D12).

In all experiments, tumor dimensions were measured once a week with a caliper and volumes calculated using the formula: D_1_ × D_2_ × D_3_/2. Tumor progression was assessed using the formula [(final volume) – (initial volume)]/(initial volume). Results were also expressed with an adapted Kaplan-Meier survival curve, using the time needed for a tumor to reach the volume of 2,000 mm^3^. The median survival was defined as the time when 50% of mice had a tumor of 2,000 mm^3^.

### Statistical analysis

A linear mixed regression model was used to determine the relationship between tumor growth and days post-graft. The fixed part of the model included variables corresponding to the number of days post-graft and the different groups. Interaction terms were built into the model. Random intercept and random slope were included to take into account the time effect. The model coefficients were estimated by maximum likelihood and considered significant at the 0.05 level. Survival rates were estimated from the xenograft date until the date when the tumor reached the volume of 2,000 mm^3^ using the Kaplan–Meier method. Median survival was presented with 95% confidence intervals. Survival curves were compared using the log-rank test. Statistical analyses were performed using the STATA 11.0 software.

### SPECT-CT imaging

### Production of ^177^Lu-labeled 3C23K

The MAb 3C23K was first conjugated with p-SCN-benzyl-DOTA, as described by Repetto-Llamazares *et al*. [59] and then labeled with 200MBq/mg ^177^Lu. ^177^LuCl_3_ was obtained from Perkin Elmer at a volumic activity of 370MBq in 8μL of 0.05M HCl and at a specific activity > 740GB q/mg. Radiochemical purity was >97% and radionuclidic purity > 99.94%. 3C23K was labeled with ^177^Lu at a specific activity of 222MBq/mg. Typically, 43 μl of 7.7 mg/ml DOTA-conjugated MAb were mixed with 40μl of 0.25M NH4OAc (pH 5.5) and pre-heated at 37°C for 5 minutes. 1.55 μl of ^177^Lu were added to the reaction mixture (222MBq/mg) and incubated at 37°C for 45 minutes. The reaction was stopped by adding 100 μl of formulation buffer (6.7mM PBS, 7.5% BSA, 1 mM DTPA, pH 7.5). Radiochemical purity was determined by thin layer chromatography (TLC) analysis using 1 μl of the reaction. Separation was done in a migration vial containing 1ml of 0.9% NaCl. The radiolabeling yield was generally above 99%. The immunoreactivity of the DOTA-conjugated antibody was compared with that of the naked antibody by flow cytometry analysis. Briefly, about 5 × 10^5^ cells were blocked in PBS/1% BSA for 30 minutes and then incubated at 4°C with 10 μg/mL DOTA-conjugated-MAbs (3C23K or R565), non-conjugated 3C23K or R565 MAbs, or PBS/1% BSA (control cells, auto-fluorescence assay) for 90 minutes. After washing, cells were incubated with FITC-conjugated anti-human IgG (0.05 μg/10^6^ cellules) at 4°C for 1 h. Cells were analyzed using a FC500 flow cytometer (Beckman Coulter).

### SPECT-CT imaging of mouse xenografts

Eight mice were xenografted with COV434-MISRII or COV434-WT cells, as described above. When tumors reached a volume of about 200 mm^3^, 250 μl of 11MBq (46 μg of 3C23K at 200MBq/mg) ^177^Lu-3C23K (direct binding study) were i.p. injected in two COV434-WT- and two COV434-MISRII-xenografted mice. Four other mice were used for the isotopic dilution study that consisted in an i.p. injection of naked 3C23K at therapeutic dose (20 mg/kg) prior to injection of ^177^Lu-3C23K.

At different time points (24, 48, 72 and 144 hours post-injection), whole-body SPECT/CT images were acquired using a four-headed NanoSPECT imager (Bioscan Inc., Washington DC). The system was equipped with a tungsten-based collimator with nine 1mm-diameter pinholes. The energy window was centered at 210 keV with ± 20% width. Acquisition times were defined to obtain 30,000 counts for each projection (generally 60–120 seconds were required) with 24 projections. The total scan time was generally around 30–60 minutes. Concurrent micro-CT whole-body images were acquired (55kV, 500 milliseconds, 240 projections) for anatomic co-registration with the SPECT data. Reconstructed data from SPECT and CT images were visualized and co-registered using Invivoscope^®^. Images and maximum intensity projections (MIPs) were reconstructed using the dedicated Invivoscope^®^ (Bioscan, Inc., Washington, USA) and Mediso InterViewXP^®^ software programs (Mediso, Budapest, Hungary). The InvivoQuant^®^ software was alternatively used for image reconstruction.

## SUPPLEMENTARY MATERIALS FIGURES


