# Supplementary material for: The anti-tumor efficacy of 3C23K, a glyco-engineered humanized anti-MISRII antibody, in an ovarian cancer model is mainly mediated by engagement of immune effector cells
Source: Oncotarget. 2017 Feb 24;8(23):37061–79. doi: 10.18632/oncotarget.15715 (PMC5513714; doi:10.18632/oncotarget.15715)
Supplement: Supplementary file 1 [file oncotarget-08-37061-s001.pdf]

The anti-tumor efficacy of 3C23K, a glyco-engineered humanized anti-MISRII antibody, in an ovarian cancer model is mainly mediated by engagement of immune effector cells

Supplementary Materials

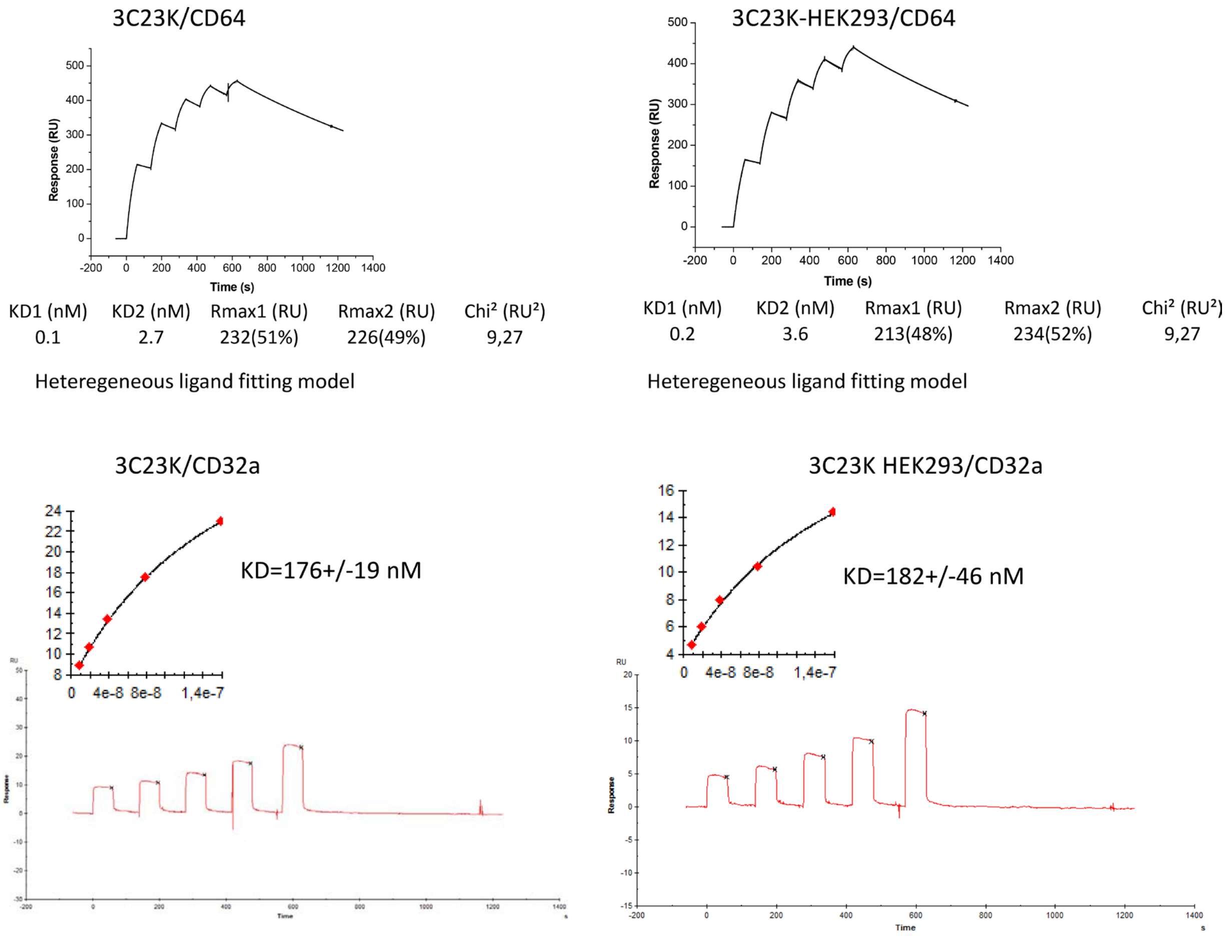

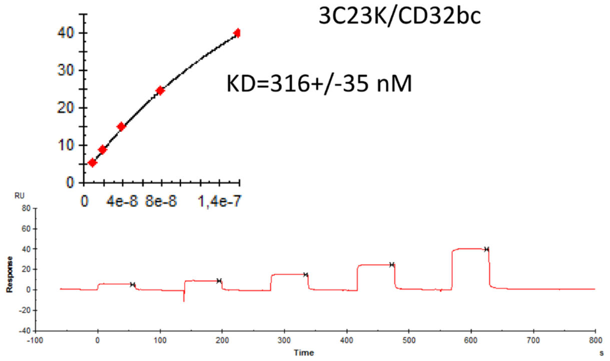

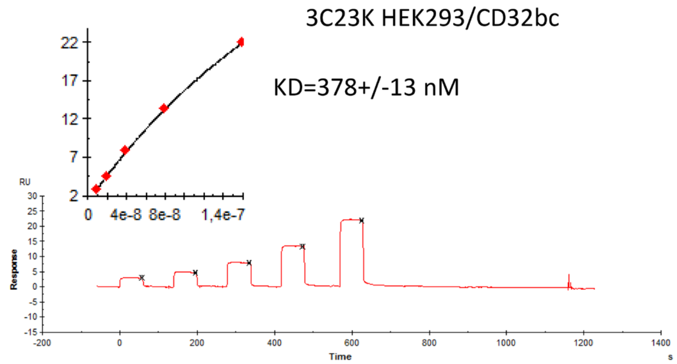

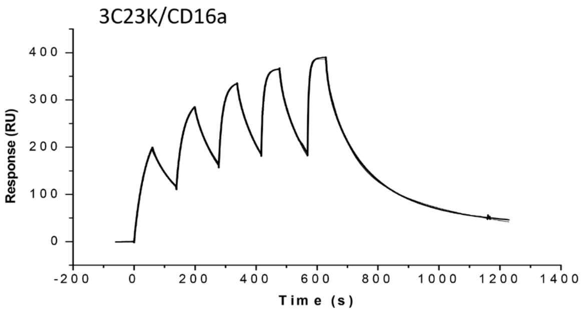

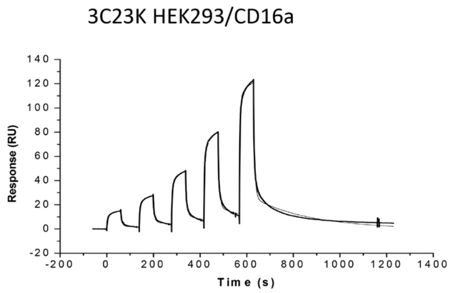

Heteregeneous ligand fitting model

KD1 (nM)	KD2 (nM)	Rmax1 (RU)	Rmax2 (RU)	Chi² (RU²)
11.8	1.08	214.8 (57%)	164.1(43%)	8,58

Heteregeneous ligand fitting model

KD1 (nM)	KD2 (nM)	Rmax1 (RU)	Rmax2 (RU)	Chi² (RU²)
164	31	166.2 (82%)	35.28 (18%)	2.41

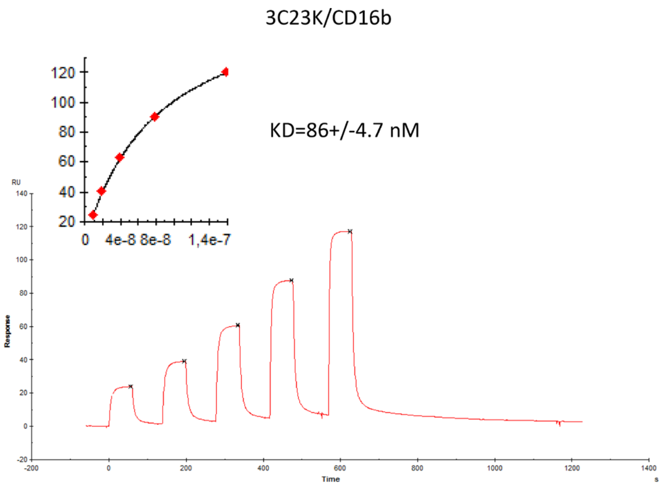

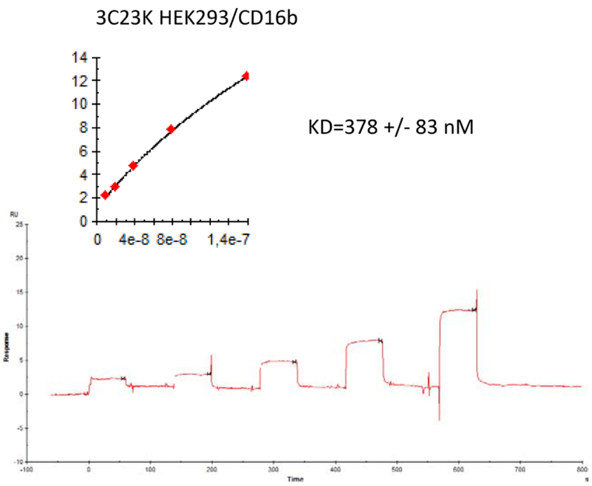

Supplementary Data: SPR fitting curves and data used for the determination of 3C23K and 3C23K-HEK293 KD values for the different human Fcγ receptors.

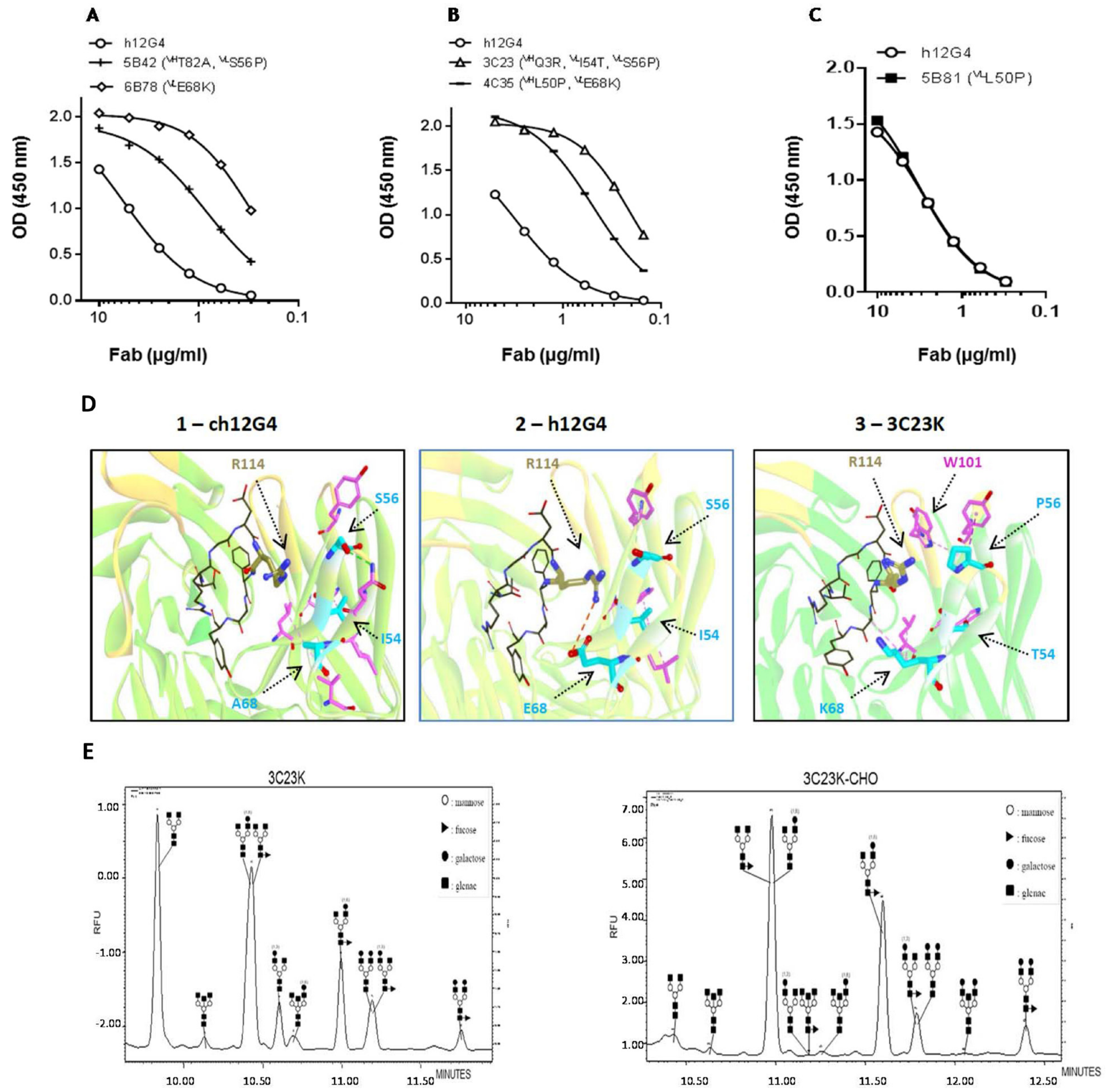

Supplementary Figure 1: Humanization of the murine antibody 12G4. (**A–C**) The Fab variants 6B78, 5B42, 4C35 and 3C23 showed a significant increase of binding affinity to immobilized recombinant MISRII-Fc fusion protein in an ELISA assay compared with humanized 12G4 (h12G4) (Figure 1D and Supplementary Figure 1A and 1B). Clones 6B78 and 4C35 shared the same mutation E68K in the VL, whereas clone 5B42 had the same mutation S56P in the VL as clone 3C23. The mutation L50P found in clone 5B81 had no effect on target binding (Supplementary Figure 1C). (**D**) Representation of the intramolecular interactions between the three residues of the CDR-L2 region that have been mutated during the affinity maturation process and the surrounding residues of the VL domain and CDR-H3 loop. The three residues of the CDR-L2 region are shown as blue sticks (I54 is partially hidden in the figure) and the other residues of the VL domain as pink sticks. The amino acids of CDR-H3 are shown as dark sticks except for the R114 residue represented in gold. The other CDR regions are highlighted in yellow in the ribbon representation. (**E**) Glycosylation profiles of 3C23K produced in YB2/0 cells (left) and in CHO-S cells (right). The two mAbs were incubated with PNGase and glycans, and analyzed by HPCE-Lif.

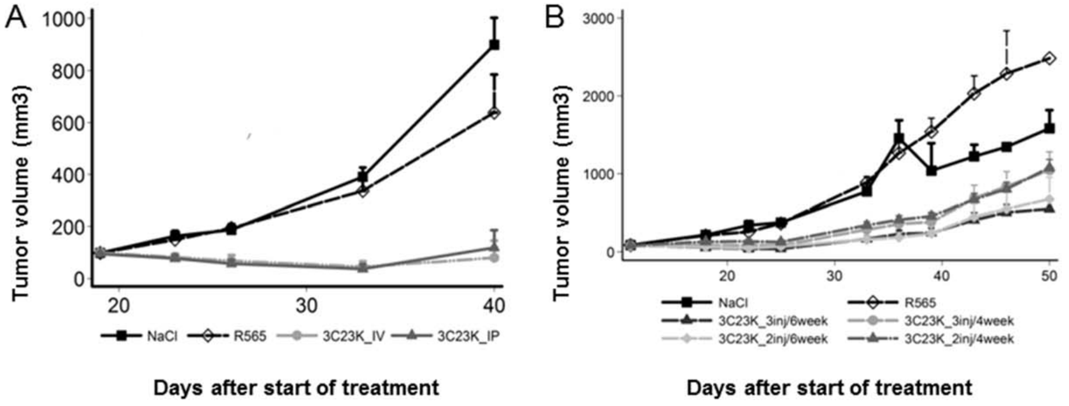

Supplementary Figure 2: *In vivo* efficacy of EMABling® 3C23K (low-fucose form) in mice xenografted with COV434- MISRII cells. (**A**) Comparison of the effect of 3C23K administered by intraperitoneal (IP) or intravenous (IV) injection relative to the irrelevant antibody R565 or NaCl, as vehicle (both IP). (**B**) Comparison of treatment schedules: 2 or 3 injections during 4 or 6 weeks. Controls (the irrelevant antibody R565 and NaCl) were administered 3 times/week for 6 weeks. Results are presented as tumor growth curves (mean and 95% CI upper bound).

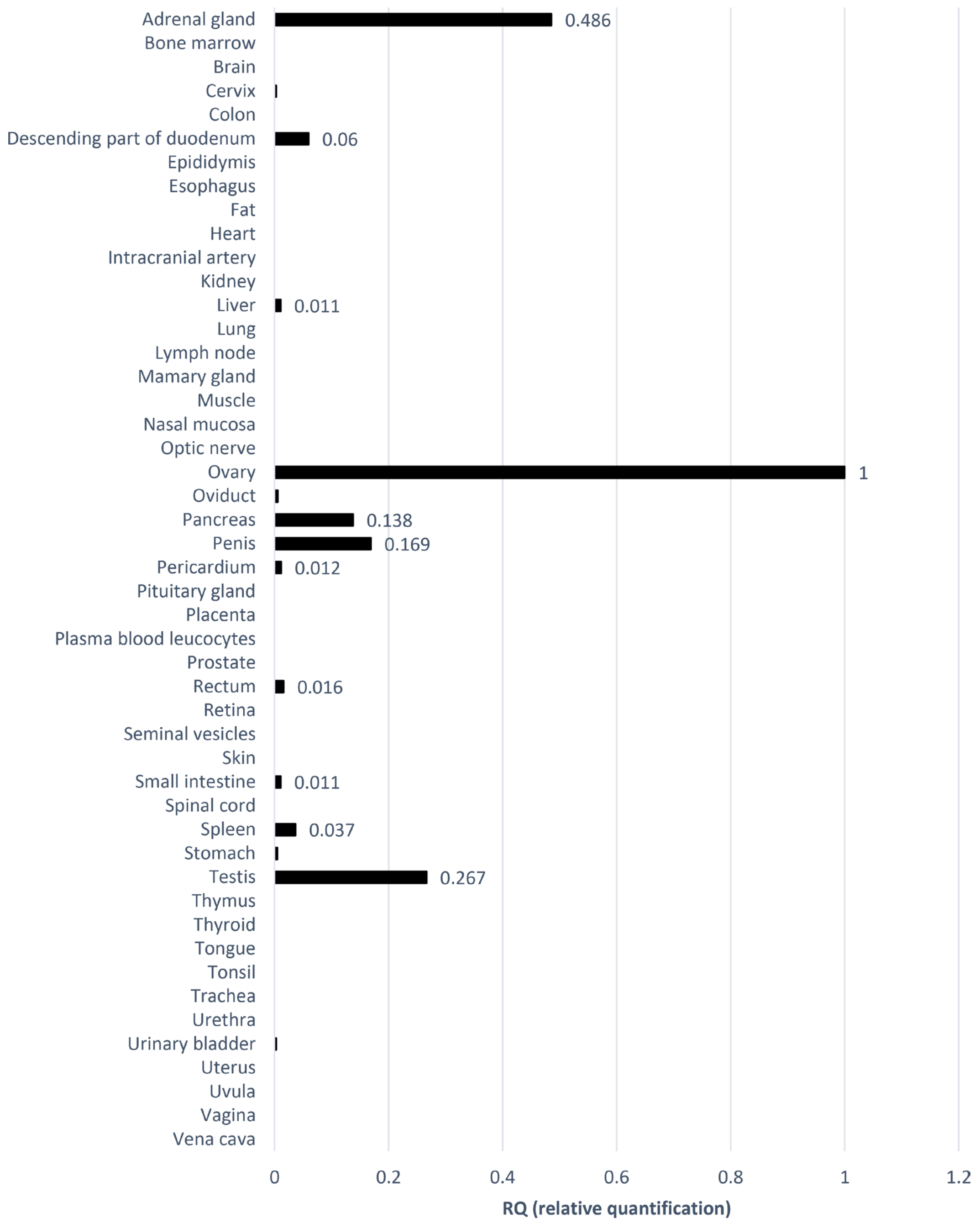

Supplementary Figure 3: qPCR analysis of MISRII expression in human normal tissues. Quantification of MISRII mRNA in a panel of 48 human normal tissues relative to the endogenous GADPH gene. Results are expressed as RQ (relative quantification) and the ovary sample was used as internal calibrator (arbitrary RQ value = 1).
